# NAD^+^-boosting agent nicotinamide mononucleotide potently improves mitochondria stress response in Alzheimer’s disease via ATF4-dependent mitochondrial UPR

**DOI:** 10.1038/s41419-024-07062-1

**Published:** 2024-10-11

**Authors:** Xi Xiong, Jialong Hou, Yi Zheng, Tao Jiang, Xuemiao Zhao, Jinlai Cai, Jiani Huang, Haijun He, Jiaxue Xu, Shuangjie Qian, Yao Lu, XinShi Wang, Wenwen Wang, Qianqian Ye, Shuoting Zhou, Mengjia Lian, Jian Xiao, Weihong Song, Chenglong Xie

**Affiliations:** 1 Department of Neurology, The First Affiliated Hospital of Wenzhou Medical University, Wenzhou, China; 2 Department of Neurology, Yuhuan City People’s Hospital, Taizhou, China; 3 The Center of Traditional Chinese Medicine, The Second Affiliated Hospital and Yuying Children’s Hospital of Wenzhou Medical University, Wenzhou, China; 4 Department of Neurology, The First People’s Hospital of Wenling, Taizhou, China; 5 Oujiang Laboratory, Wenzhou, Zhejiang China; 6 School of Pharmaceutical Science, Wenzhou Medical University, Wenzhou, China; 7 Key Laboratory Of Alzheimer’s Disease Of Zhejiang Province, Institute Of Aging, Wenzhou Medical University, Wenzhou, Zhejiang China; 8 Department of Geriatrics, Geriatric Medical Center, The First Affiliated Hospital of Wenzhou Medical University, Wenzhou, Zhejiang China

**Keywords:** Cellular neuroscience, Alzheimer's disease

## Abstract

Extensive studies indicate that mitochondria dysfunction is pivotal for Alzheimer’s disease (AD) pathogenesis; while cumulative evidence suggests that increased mitochondrial stress response (MSR) may mitigate neurodegeneration in AD, explorations to develop a MSR-targeted therapeutic strategy against AD are scarce. We combined cell biology, molecular biology, and pharmacological approaches to unravel a novel molecular pathway by which NAD^+^-boosting agent nicotinamide mononucleotide (NMN) regulates MSR in AD models. Here, we report dyshomeostasis plasma UPR^mt^-mitophagy-mediated MSR profiles in AD patient samples. NMN restores NAD^+^ metabolic profiles and improves MSR through the ATF4-dependent UPR^mt^ pathway in AD-related cross-species models. At the organismal level, NAD^+^ repletion with NMN supplementation ameliorates mitochondrial proteotoxicity, decreases hippocampal synaptic disruption, decreases neuronal loss, and brain atrophy in mice model of AD. Remarkably, omics features of the hippocampus with NMN show that NMN leads to transcriptional changes of genes and proteins involved in MSR characteristics, principally within the astrocyte unit rather than microglia and oligodendrocytes. In brief, our work provides evidence that MSR has an active role in the pathogenesis of AD, as reducing mitochondrial homeostasis via *atf4* depletion in AD mice aggravates the hallmarks of the disease; conversely, bolstering mitochondrial proteostasis by NMN decreases protein aggregation, restores memory performance, and delays disease progression, ultimately translating to increased healthspan.

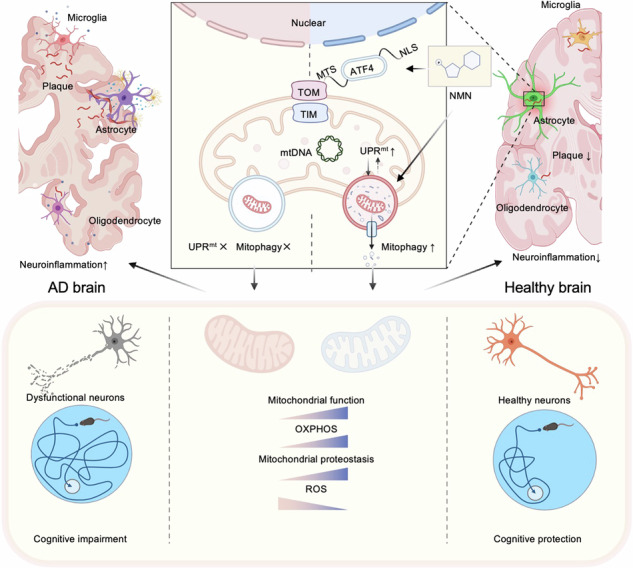

## Introduction

Mitochondria are a hub for the cellular metabolism of most eukaryotic cells that provide the necessary energy for a variety of different processes and, as such, orchestrate many metabolic reactions, including the electron transport chain, Krebs cycle, and fatty acid β-oxidation et al. [1]. Damaged mitochondria have surfaced as one of the most discussed central players in Alzheimer’s disease (AD) and thus may act as a “risk factor” for AD occurrence [2]. In addition, mitochondrial maintenance and dynamics undergo age-dependent structural and functional decline, a hallmark of aging and age-related diseases [3]. Among them, AD is a complicated, multifactorial, progressive neurodegenerative disease, and mitochondrial malfunction is supposed to be a common pathological characteristic [4]. Abnormalities of mitochondria can be observed systemically in AD-affected subjects, presenting decreased mitochondrial respiration activity, perturbations in mitochondria quality control, and alterations in morphology [5]. Historically, plentiful progress has been made, and genetic and biochemical studies in cross-species models now hint that mitochondrial dysfunction and cellular energy exhaustion are critical to AD progression [6].

In this respect, recent studies have shown that a conserved mitochondrial stress response (MSR) signature is present in diseases concerning amyloid-β (Aβ) proteotoxicity that involves the mitochondrial unfolded protein response (UPR^mt^) and mitophagy pathways [7]. Increasing mitochondrial proteostasis by pharmacologically and genetically targeting MSR to delay Aβ proteotoxic diseases in worms. Similar approaches could be reasonably translated into treatments for AD. To maintain mitochondria homeostasis under stress, cells sense and respond to injured mitochondrial by activating a program known as the UPR^mt^ [8], which comprises the elicitation of mitochondrial chaperones assisting in appropriate protein folding and of proteases expediting clearance of misfolded proteins [9]. Indeed, a few studies suggested the role of UPR^mt^ in AD background [10, 11]. Under physiological or disease conditions, UPR^mt^ and mitophagy are the two significant mitochondrial quality control pathways that cooperate with stress resistance, as both are responsive to similar forms of mitochondrial stress [12]. To keep mitochondrial networks and functions intact, cells have developed quality control systems that get rid of damaged or superfluous mitochondria by UPR^mt^ and mitophagy [13]. Recently, our group provided comprehensive evidence that mitophagy was impaired in the hippocampus of AD patients, in induced pluripotent stem cell (iPSC)-derived AD human neurons, and in animal AD models. Small compound mitophagy inducers abolished AD-related neuropathology phenotypes and relieved memory impairment in multidimensional AD models [14, 15]. However, it is still unclear whether mitochondrial impairments are prime culprit disease drivers and whether boosting mitochondrial biogenesis provides neuroprotection in AD.

Nicotinamide adenine dinucleotide (oxidized form, NAD^+^) is a crucial cellular cofactor involved in over 500 enzymatic reactions linked to metabolism, mitochondrial health, stem cell rejuvenation, and longevity in worms, Drosophila, and mice [16–18]. However, little is known about NAD^+^ metabolism and the levels of MSR in AD patients’ blood. Here, utilizing AD patients’ blood samples, AD-related cells, and transgenic animal models of AD, we propose that defective MSR induces the accumulation of damaged mitochondria, promoting AD pathology and memory loss, and improving mitochondrial homeostasis from the nucleus to the mitochondria through UPR^mt^-mitophagy pathways by pharmacologically NAD^+^-booster is an effective therapeutic strategy. Given the persistent failures in anti-AD drug discovery, approaches focusing on the broader aspects of the AD pathology profile, such as defective MSR, may hold a promising prospect.

## Results

### Disturbed plasma UPR^mt^-mitophagy mediated MSR profiles in AD subjects

**Fig. 1:**
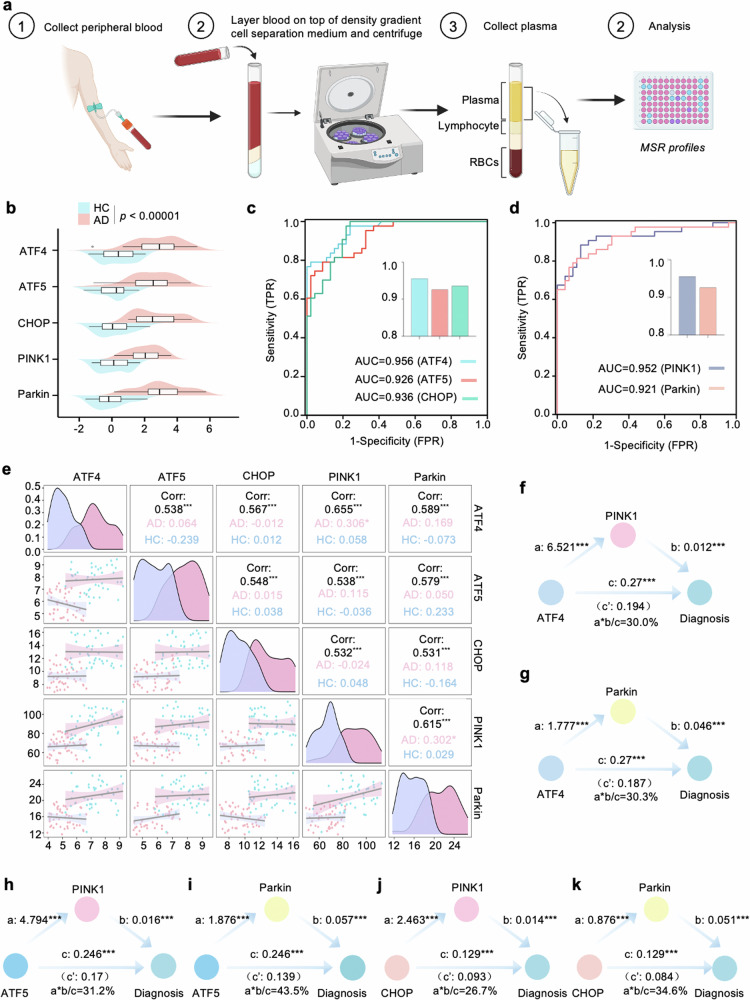
Plasma UPR^mt-^mitophagy mediated MSR profiles in AD subjects. **a** Schematic of plasma MSR indicators examination for every individual enrolled in the study. The recruitment eligibility assessment led to the inclusion of 43 AD and 46 HC subjects. **b** The distribution of MSR blood biomarker levels across AD and HC individuals is illustrated by the mountain map. **c**, **d** Receiver operating characteristic (ROC) analysis was performed to assess the ability of plasma UPR^mt^ and mitophagy proteins to distinguish between AD and HC, and the area under the curve (AUC) value is presented. **e** A multivariate correlation scatter matrix graph was used to examine the relationships among the MSR blood biomarkers in both Alzheimer’s disease (AD) and healthy control (HC) participants. **f**–**k** Schematic illustration presents the estimated proportion of the association between plasma UPR^mt^ levels and AD risk that is mediated by mitophagy blood biomarkers. Data are shown as mean ± s.e.m. The *p*-values are indicated on the graphs. ns not significant.

Research on the biology of MSR has been gaining momentum in age-associated diseases. See Fig. 1a for a flowchart of the plasma assay process. A total of 91 participants were selected, comprising 46 individuals with healthy control (HC), and 43 clinically diagnosed with AD. The basic information includes average age, sex ratio, height, weight, BMI, education, disease duration, CDR, MoCA, MMSE, and the levels of MSR proteins provided. Of note, there were no statistical differences in age, sex ratio, Height, and education between AD and HC, which indicated that the subjects in each group were comparable. Details for the basic characteristics of these subjects are provided in Supplementary Tables 1–3. The levels of plasma MSR profiles, including ATF4, ATF5, CHOP, PINK1, and Parkin were significantly higher in AD compared to the HC group (Fig. 1b). Meanwhile, a high ATF4 and PINK1 concentrations were observed in serious memory status in terms of clinical dementia rating (CDR) scores (Supplementary Fig. 1a). Assessing the utility of MSR-UPR^mt^ levels to discriminate between clinically defined AD and HC, we found an area under the ROC curve (AUC) of 0.956 (95% CI: 0.920–0.991) for ATF4, 0.926 (95% CI: 0.875–0.976) for ATF5, and 0.936 for CHOP (95% CI: 0.891–0.982; Fig. 1c). Similarly, in terms of MSR-mitophagy markers, ROC tests compared AD patients against the HC group (Fig. 1d), and the AUC for PINK1 was 0.952 (95% CI: 0.861–0.982) and 0.921 (95% CI: 0.857–0.977) for Parkin, indicating MSR is a suitable diagnosis marker panel. We analyzed the correlation between UPR^mt^ markers (ATF4, ATF5, and CHOP) and mitophagy indexes including PINK1 and Parkin. Based on the Spearman correlation coefficients (*ρ*-values), we found plasma UPR^mt^ markers positively correlated with mitophagy indexes in whole groups (Fig. 1e), indicating that plasma UPR^mt^ markers and mitophagy proteins have similar and related expression trajectories. Then, to explore whether the relationships of UPR^mt^ with AD diagnosis were mediated by mitophagy proteins, mediation analyses with 10,000 bootstrapped iterations were carried out to examine the effects based on the method proposed by Baron and Kenny [19]. The results demonstrated that the relationship between ATF4 and AD was mediated by PINK1 and Parkin with an approximate proportion of mediation of 30.0% (*p* < 0.001) and 30.3% (*p* < 0.001), respectively (Fig. 1f, g). Consistent with these results, we found that the association between ATF5/CHOP and AD diagnosis was also partially mediated by PINK1 and Parkin (Fig. 1h–k).

### Activation of the NAD^+^-UPR^mt^ pathway is beneficial for AD-related cell models

**Fig. 2:**
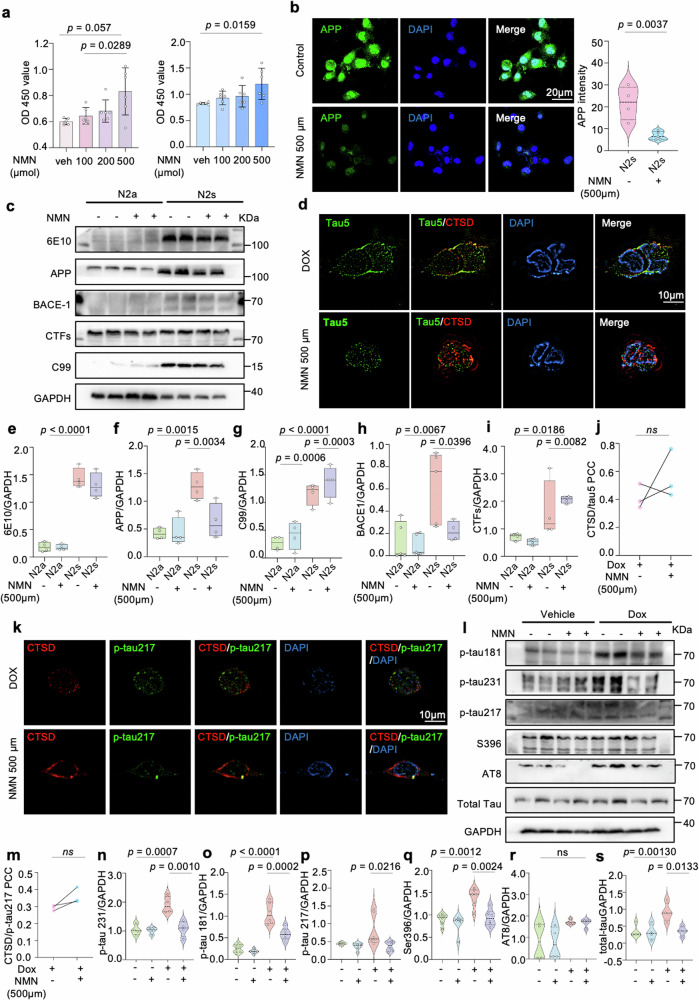
NAD^+^ supplement slashes hyperphosphorylated tau and Aβ peptide in vitro. NMN restores Aβ expression in N2a APPswe cells and inhibits tau hyperphosphorylation in HEK293 cells Tau P301L after supplementing with either VEH or 500 μm NMN for 24 h. **a** Cell viability results of N2a mouse neuroblastoma cells and overexpressing Swedish K595N and M596L mutations cells depicting a dose-dependent NMN effect. Data were analyzed by one-way ANOVA followed by Sidak’s multiple comparisons test. **b** Representative confocal images showing the APP level of the NMN-treated N2s cells. Data were analyzed by a two-sided unpaired *t*-test. Scale bar, 25 μm. **c**, **e**–**i** SDS-PAGE, and corresponding quantification of APP and its indicated protein expressions indicating NMN effects. Data were analyzed by two-sided one-way ANOVA followed by Tukey’s multiple comparisons test. **d**, **j**, **k**, **m** Representative 3D reconstruction images and corresponding quantification of colocalization of Tau5, tau-Thr^217^ and CTSD. Scale bar, 10 μm. **l**, **n**–**r** Effects of NMN on the expression levels of different p-Tau sites (Thr181, Thr231, Thr217, AT8, and Ser396) in HEK293 cells Tau P301L. **s** The total Tau level change between NMN-treated and non-treated group. Data were analyzed by two-sided one-way ANOVA followed by Tukey’s multiple comparisons test. At least three experiments were repeated independently with similar results. Full scans of all the blots are in the Supplementary Data. All experiments were performed independently with at least three biological replicates. Data are shown as mean ± s.e.m. The *p*-values are indicated on the graphs. ns not significant.

We wondered whether a reduction of NAD^+^ levels drove the accelerated pathological phenotypes in AD [20]. We increased cellular NAD^+^ levels by incubating the Aβ (N2s: N2a mouse neuroblastoma cells expressing the Swedish K595N and M596L mutations in Amyloid-beta precursor protein) and tau cells (HEK293 cells Tau P301L) with NAD^+^ precursor nicotinamide mononucleotide (NMN, 0.5 mM) for 24 h. NMN increased the cell viability in a dose-dependent manner (Fig. 2a) and reduced the BACE-1 and 6E10 fluorescence signal intensities (Fig. 2b and Supplementary Fig. 1b–e). Moreover, NMN lowered the protein levels of 6E10 (full-length APP, C99), BACE-1, and CTFs in mouse neuroblastoma N2s cells (Fig. 2c, e–i), suggesting a possibility of amelioration in Aβ pathology of NMN by inhibition of BACE-1 activity. These results were further enhanced by the transcriptional level evidence that NMN treatment resulted in decreased mRNA levels of APP and BACE-1 (Supplementary Fig. 1f). Regarding tauopathy in vitro by using mammalian HEK293 cells expressing pTRE3G-cherry-BI promoter-EGFP Tau P301L, NMN treatment offset the tau injury and resulted in a decrease in the expression of several phosphorylated-tau (p-tau) sites, including thr181, thr231, thr217, and ser396 (Fig. 2d, j–s, and Supplementary Fig. 1g). Interestingly, 3D reconstructed imaging showed that more tau and lysosome (CTSD marker) colocalized in the NMN-treated cells (Fig. 2d, k), which indicates that cellular tau pathology is probably relieved by NMN treatment through autophagy.

**Fig. 3:**
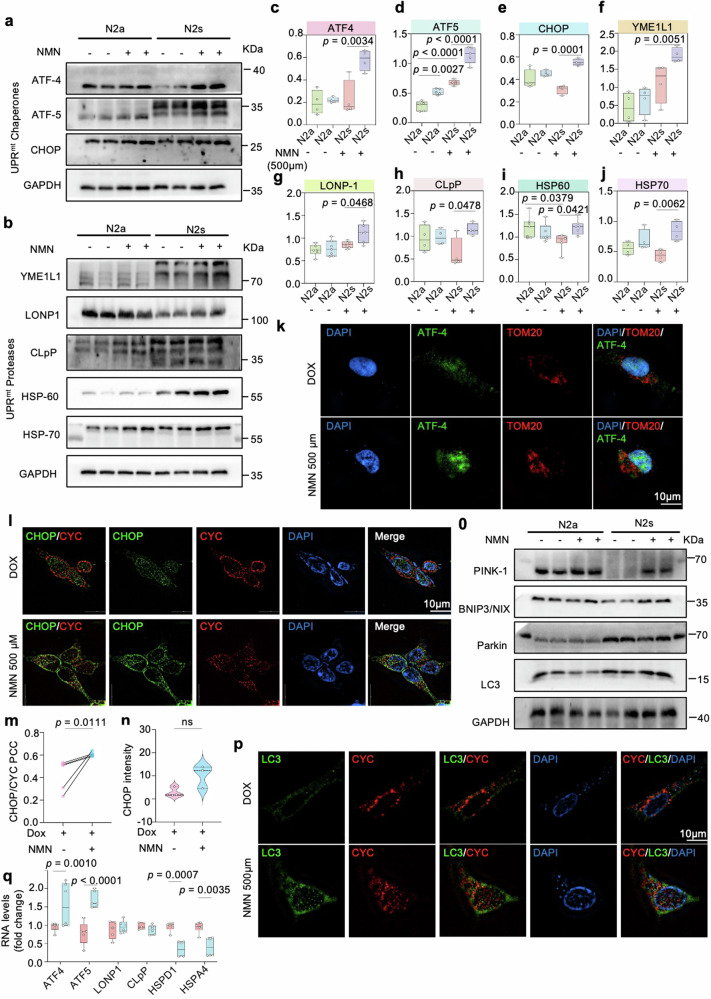
MSR signature enhanced by NAD supplementation in multiple cell models. **a**–**j** Levels of UPR^mt^-related chaperone and protease proteins in N2a APPswe cells (*n* = 6 per group). At least three experiments were repeated independently with similar results. Data were analyzed by two-sided one-way ANOVA followed by Tukey’s multiple comparisons test. **k** Confocal images of HEK293 Tau P301L cells double immunostained with ATF4 (green) and mitochondria outer membrane protein TOM20 (red) antibody, after 24 h of NMN incubation. Scale bar, 10 μm. **l**–**n** 3D reconstruction images of HEK293 Tau P301L cells double immunostained with CHOP (green) and CYC (red) antibodies. Scale bar, 10 μm. **o** Relative levels of proteins implicated in the mitophagy pathway. **p** Quantification of the colocalization of the mitochondrial protein CYC and the autophagy protein LC3 in HEK293 Tau P301L cells after treatment with NMN. Scale bar, 10 μm. Immunoblot data were analyzed by two-sided one-way ANOVA followed by Tukey’s multiple comparisons test while the PCC were analyzed by two-sided unpaired *t*-test. **q** UPR^mt^ transcript levels in N2a neuroblastoma cell line. All experiments were performed independently with at least three biological replicates. Data are shown as mean ± s.e.m. The *p*-values are indicated on the graphs. ns not significant.

We speculated that one of the main contradictions in AD could be caused by mitochondrial dysfunction and evaluated a series of mitochondrial stresses that have been linked to the decline of intracellular NAD^+^ levels [6, 21]. The UPR^mt^-mitophagy pathways are also possibly induced by NAD^+^-boosting compounds, such as NR, and Olaparib, in worms and mammalian tissues [22]. Thus, we further examined the impact of the generally identified NAD^+^ stimulators NMN on conserved MSR involving the UPR^mt^ and mitophagy pathways. NMN was recently supposed to be a bona fide NAD^+^ precursor in vertebrates, effectively augmentation of NAD^+^ concentration in various tissues and indirectly quantifiable in plasma [23]. Notably, MSR was prominently improved in NMN-treated N2s cells, as shown by increased UPR^mt^ chaperone proteins, such as ATF4, ATF5, and CHOP (Fig. 3a, c–e), as well as UPR^mt^ protease proteins (e.g., YME1L1, LONP1, CLpP, HSP60, and HSP70) (Fig. 3b, f–j), presumably to maintain protein homeostasis within mitochondria. To duplicate the validation of the NMN effects on the UPR^mt^ pathway in vitro AD model. We performed immunostaining, quantifying ATF4, ATF5, CHOP, and HSP70 markers for UPR^mt^ activity [8]. Consistently, ATF4, ATF5, CHOP, and HSP70 signal intensities were significantly elevated after NMN treatment (Fig. 3k–n and Supplementary Fig. 2a–f). Of note, the results showed that ATF4, ATF5, and CHOP mainly colocalized with the nucleus (DAPI) compared to the mitochondrion (TOM20, CYC), indicating UPR^mt^ chaperones traffic to the nucleus through NLS (nuclear localization sequence) to activate the UPR^mt^ response under this condition (Fig. 3k–n and Supplementary Fig. 2a, c–f).

The UPR^mt^ and mitophagy can be activated simultaneously when mitochondria suffer from stresses [24]. Hence, we further scrutinized the mitophagy-inducing capacity of the NMN by examining a list of well-known mitophagy proteins critical in mitochondrial metabolism [25]. As expected, NMN increased protein levels of PINK1, Parkin, BNIP3/NIX, and LC3 expressions (Fig. 3o and Supplementary Fig. 2h), a very similar pattern was seen in the immunostaining method of PINK1 (Supplementary Fig. 2g). The percentage of PINK1 colocalized to the lysosome (LAMP2) was higher compared to the nucleus (Supplementary Fig. 2g, i), indicating mitophagy induction. Moreover, the signal of LC3, an autophagosome marker in mammals, was higher with NMN treatment and primarily colocalized to the mitochondria (Fig. 3p and Supplementary Fig. 2j). Here, we extended the analysis in mRNA latitude and found NMN notably upregulates several UPR^mt^ transcripts, especially *atf4* and *atf5* (Fig. 3q); however, no effects on the mitophagy transcript genes were observed (Supplementary Fig. 2k). All these data manifested boosting NAD^+^ levels with NMN appeared to improve MSR through the upregulation and/or activation of UPR^mt^ and mitophagy pathways in AD cell models.

### Restoration of the NAD^+^-UPR^mt^ signaling ameliorates mitochondrial homeostasis in AD cells

A variety of core events in apoptosis and oxidative stress focus on mitochondria, including the release of caspase activators, changes in electron transport, and loss of mitochondrial transmembrane potential et al. [26]. Since NMN enhanced the MSR profiles in AD cell models, we further asked whether NMN could protect against apoptosis and oxidative stress. We found that NMN had a statistically significant effect on the reduction of the Bax/Bcl-2 ratio and increased the SOD1/2 levels (Supplementary Fig. 3a–f). Moreover, immunofluorescence analysis showed significantly decreased levels of Bax and Caspase-3 with NMN administration (Supplementary Fig. 3g–j), suggesting apoptosis and oxidative stress were suppressed in the context of NAD^+^- augmentation. Of note, it has been reported that damaged mitochondria and necroptosis, a programmed form of necrosis, were observed in aging and in postmortem AD brains [27, 28]. To determine whether necroptosis was inactivated in AD mice with NAD^+^ augmentation, we found that NMN indeed inhibited necroptosis, as evidenced by an apparent reduction in RIPK1, RIPK3, and MLKL expressions by immunoblotting analysis (Supplementary Fig. 3k, l).

Pharmacologically, supplementation of NMN in Tau P301L cells significantly relieves mitochondrial homeostasis in terms of total mitochondria contents compared with vehicle treatment (Supplementary Fig. 4a). These observations were confirmed at the molecular level by a higher mitochondrial DNA-encoded MTCO1 to nuclear DNA-encoded ATP5a ratio (Supplementary Fig. 4b), suggesting NMN-induced mito-nuclear protein imbalance. To verify that AD-like pathology phenotype amelioration by NMN is dependent on the ATF4 pathway, we generated two *atf4* siRNA loci for this set. We performed immunoblotting and immunostaining for Aβ pathology and found that NMN-induced low levels of 6E10, C99, and APP in the N2s cell were dependent on the ATF4 signal (Supplementary Fig. 4c, d). Analogously, ATF4 knockdown was also sufficient to block the induction of mito-nuclear protein imbalance with a relative decrease in the ratio between ATP5a and MTCO1 in N2s cells (Supplementary Fig. 4e), representing an epistatic link between NAD^+^-UPR^mt^ signaling and mitochondrial homeostasis in vitro.

### NAD^+^ supplementation inhibits memory loss and forestalls neuropathology phenotypes in 5xFAD mice

**Fig. 4:**
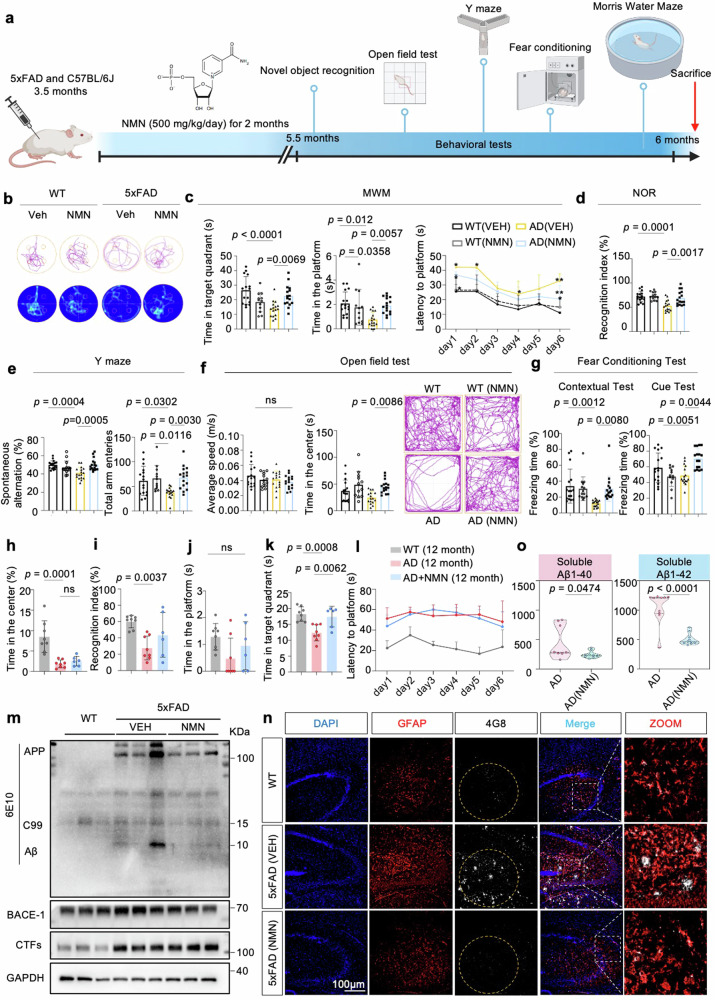
NAD^+^ booster ameliorates cognitive decline and Aβ deposition in 5xFAD mice. **a** A workflow diagram of NMN (500 mg/kg, intraperitoneal injection) treatment began at 3.5-month-old mice for 2 months. **b**–**g** The behavioral test results of 6-month-old mice. Data were analyzed by two-sided one-way ANOVA followed by Tukey’s multiple comparisons test. **b** The representative swimming tracks of mice from a probe trial day (*n* = 16 mice in WT (VEH), *n* = 16 mice in AD (VEH), *n* = 13 in WT (NMN), and *n* = 16 in AD (NMN)). **c** Spatial memory analysis of each group. Left, time in the target quadrant in the probe trial; center, platform frequency of mice passed through the platform in the probe trial at day 7; right, latency to escape to a hidden platform in the MWM during a 6-day training period. Data were analyzed by two-way ANOVA followed by Tukey’s multiple comparisons test. **d**–**g** New object recognition (**d**), Y maze (**e**), Open Field test (**f**), and contextual and cued fear conditioning (**g**) tests were performed. **h**, **i** The behavioral tests analysis as in (**b**–**g**) in 12-month-old mice (*n* = 13 mice in WT (VEH), *n* = 7 mice in AD (VEH), and *n* = 7 in AD (NMN)). New object recognition (**i**) and open Field test (**h**) tests were performed. **j**–**l** The results of MWM in each group in 12-month-old mice. Data were analyzed by one-way ANOVA followed by Tukey’s multiple comparisons test for (**h**–**k**), while two-way ANOVA was used for (**l**). **m** Effects of NMN on the expression levels of APP and its indicated metabolites, BACE-1, and CTFs hippocampal tissues from 5xFAD. Full-length APP, C99, and Aβ were detected with 6E10 antibody (*n* = 6 mice per group; one-way ANOVA). **n** Confocal images of 4G8+ GFAP+ double-positive cells from hippocampi of 6-month-old WT, 5xFAD and 5xFAD (NMN) mice. Insets were enlarged from hippocampal areas. Scale bar, 100 μm. The experiments were repeated from three independent experiments with the results shown as mean ± s.e.m. **o** Soluble and insoluble Aβ_1–40_ and Aβ_1–42_ levels in hippocampal tissues (*n* = 9 mice; two-sided unpaired *t*-test). All experiments were performed independently with at least three biological replicates. Data are shown as mean ± s.e.m. The *p*-values are indicated on the graphs. ns not significant.

To investigate the effect of NAD^+^ supplementation on behaviors in 5xFAD mice (Fig. 4a), we performed the Morris water maze (MWM) test to measure spatial learning and memory capacity. In the learning session, the results indicated that NMN treatment significantly improved the learning abilities of 5xFAD mice during the training trial. Moreover, 5xFAD with NMN mice displayed shorter latency and swimming distance entry to the hidden target platform, more platform frequency, and more time spent in the target quadrant during the probe trial compared to the AD mice (Fig. 4b, c), indicating better memory retention in the NMN-treated mice. The average speed was not different between the two groups, representing normal motor functions. Additionally, a novel object recognition (NOR) test was performed to examine mice’s visual recognition memory and cognitive ability. Consistent with the MWM results, the NOR test showed that NMN-treated mice preferred to touch the novel object compared to the NMN-free mice group (Fig. 4d), further suggesting better cognition in 5xFAD-NMN treatment mice. Similarly, NAD^+^ replenishment improved the performance of spatial memory in 5xFAD mice using the Y maze for spontaneous alternations and total arm entries (Fig. 4e). Moreover, NMN-treated mice showed more time in the center zone (Fig. 4f), showing their locomotor activity is normal and NMN has a mild effect on anxiety. Finally, NMN also increased context-dependent and cued-dependent memory in the mice with 5xFAD, which was impaired compared to WT mice, as assessed by fear conditioning (Fig. 4g), indicating a potentially positive impact on cognitive function. Together, these behavioral tests demonstrate that NMN significantly positively affects cognitive impairment in AD mice at 6 months of age.

Then, to address the effect of NMN on aged AD mice, 9-month-old 5xFAD mice were administered NMN for three months [29]. However, in this issue, pharmacological supplementation of NMN cannot abrogate Aβ-induced memory deficits in 12-month-old 5xFAD mice even though there exists a tendency, as seen in the NOR, OFT, and MWM (Fig. 4h–l), consistent with what was observed in the AD frustrated drug clinical trials. Many explanations have been proposed for the failures of AD trials, including starting the therapies too late [30]. We next aimed to confirm the relevance of AD pathologies and NAD^+^-boosting in 5xFAD mice. Supplementation with NMN ameliorates the Aβ process-related proteins, including 6E10 (Full-length APP, C99, and Aβ), BACE-1, and CTFs in the hippocampus (Fig. 4m). Consistently, immunofluorescence analysis revealed that Aβ plaque burden (4GB) was diminished in the 4G8+ GFAP+ double-positive cells in the hippocampus with NMN treatment compared to the 5xFAD-vehicle group (Fig. 4n and Supplementary Fig. 5a–c). We further characterized amyloid deposition in the hippocampus of 5×FAD mice and found that NMN markedly decreased APP-immunopositive dots compared to the vehicle group (Supplementary Fig. 5d). Moreover, several other common features of AD pathology, including soluble levels of Aβ_1–40_ and Aβ_1–42_ were diminished after NMN treatment (Fig. 4o). While astrocytic and microglia activation, measured by GFAP and Iba1 levels (Supplementary Fig. 5e, f), were also decreased in the NMN-treated AD mice compared with vehicle-treated. These findings in transgenic mouse models of AD suggest a robust neuroprotective role of NMN against AD pathologies and cognitive deficits.

### MSR signature can be improved by increased NAD^+^ biosynthesis in mice

**Fig. 5:**
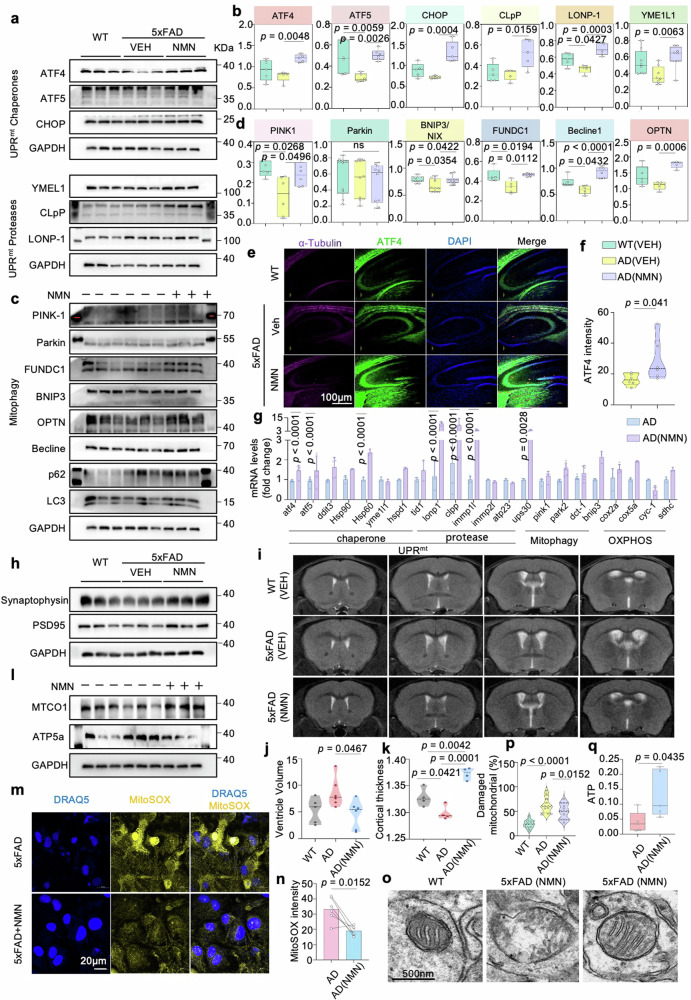
Cross-species MSR signature attenuates mitochondrial dysfunction. **a**, **c** Effects of NMN on the expression level of proteins involved in UPR^mt^ and mitophagy in the 5xFAD mice hippocampi of individuals with and without NMN (*n* = 3 biologically independent samples; two-sided unpaired *t*-test). **b**, **d** Corresponding quantification of western blot data of (**a**, **c**) (*n* = 3 mice in each group). Data were analyzed by two-sided one-way ANOVA followed by Tukey’s multiple comparisons test. **e**, **f** Representative immunostained images (**e**) and quantification (**f**) of ATF4 in the hippocampi of AD (VEH) and AD (NMN) tissues (*n* = 3 mice; two-sided unpaired *t*-test). Scale bar, 100 μm. **g** The mRNA expression measurement of MSR signature after supplementing with NMN in 5xFAD mice (*n* = 3; two-way ANOVA). **h** Synaptic proteins from the 6-month hippocampi were analyzed by Western blotting (*n* = 3 mice in each group). Data were analyzed by two-sided one-way ANOVA followed by Tukey’s multiple comparisons test. **i**–**k** we obtained T2-weighted anatomical images to analyze the structure difference in the ventricle system using a 9.4 Tesla magnetic resonance imaging (MRI) scanner. Data were pooled from at least 3 biological replicates. Data were analyzed by two-sided one-way ANOVA followed by Tukey’s multiple comparisons test. **l** Mito-nuclear protein imbalance evaluated by the ratio of mitochondrial DNA (mtDNA)-encoded protein (MTCO1) and nuclear DNA (nDNA)-encoded protein (ATP5a) in three groups (*n* = 3 mice; one-way ANOVA). **m**, **n** Representative immunostaining and quantification of the MitoSOX in the cultured primary astrocytes (*n* = 6 mice; two-sided unpaired *t*-test). Scale bar, 20 μm. **o**, **p** Representative electron microscopic images, and multiple quantifications, showing the effects of NMN on mitochondrial morphology in mouse hippocampal brain tissues. The quantification was obtained in two independent experiments performed in duplicates in at least 20 ROIs. **q** ATP Assay was performed in 6-month-old AD mice brain (*n* = 5 mice, two-sided unpaired *t*-test). All experiments were performed independently with at least three biological replicates with similar results. Data are shown as mean ± s.e.m. The *p*-values are indicated on the graphs. ns not significant.

We next measured UPR^mt^ and mitophagy pathways as the two major mitochondrial quality control tactics for MSR in the mouse hippocampus to evaluate whether their expression is also co-regulated with NMN in the AD model. We have previously found that several UPR^mt^ and mitophagy pathway proteins were upregulated during NMN background in vitro. Here, we extend that analysis in vivo and observe that, compared to 5xFAD mice without NMN treatment, several UPR^mt^ chaperone proteins (ATF4, ATF5, and CHOP), UPR^mt^ protease proteins (YME1L1, CLpP, and LONP1), and mitophagy targets (PINK1, Parkin, FUNDC1, OPTN, and Becline 1) were upregulated in the hippocampus after NMN supplementation (Fig. 5a–d), similar to what has previously been shown in AD cell models, presumably to maintain protein homeostasis within mitochondria in mice brain. In addition, consistent with our immunoblotting results, immunostaining showed that ATF4 levels were significantly increased in the NMN-treated 5xFAD mice compared to 5xFAD-vehicle mice (Fig. 5e, f). To validate these results, we then investigated the underlying transcriptional levels of MSR in the mouse hippocampus and found NMN increased the mRNA levels of *atf4*, *atf5*, *hsp60*, *lonp1*, *clpp*, and *immp1l* while having no significant effect on *pink1* and *parkin* (Fig. 5g). Collectively, the induction of the MSR under NMN administration was observed at the protein and transcriptional levels in cross-species AD models.

### NMN ameliorates hippocampal synaptic disruption and decreases neuronal loss and brain atrophy

Synapse degeneration is supposed to be an intermediate step and a vital pathophysiological hallmark of AD. Emerging evidence indicates that a maladjustment in the number and function of synapses, which occurs later than Aβ accumulation and relates to disease progression [31]. Thus, we investigated synaptic alterations in 5xFAD mice with and without NMN. Biochemical analysis by fractionation of synaptosomes from 5xFAD-NMN mice hippocampus demonstrated a significant increase in levels of synaptophysin and PSD-95 (Fig. 5h and Supplementary Fig. 5g, h), pre- and post-synaptic markers, respectively. Moreover, NMN also reversed the reduction in hippocampal dendritic spine density by the Golgi staining (Supplementary Fig. 5i), indicating synaptic disruption retention with NMN administration. However, immunofluorescence staining of NeuN-positive cells displayed comparable intensity in the NMN-treated 5xFAD mouse brains (Supplementary Fig. 5j). Consistent with neuronal loss and brain atrophy, AD patients exhibit enlarged ventricles. In our experiments, we obtained T2-weighted anatomical images to analyze the structure difference in the ventricle system using a 9.4 Tesla magnetic resonance imaging (MRI) scanner. Increased volume of the inferior lateral ventricle has been described as an early imaging marker for AD, we next explore the lateral ventricle changes in our AD mouse model [32]. In coronal sections, an enlargement of the lateral ventricles and thinner cortical thickness were visible in 12-month-old 5xFAD mice compared with WT littermates; notably, these discrepancies can be offset by the NMN treatment (Fig. 5i–k).

Remarkably, immunoblotting of hippocampi homogenates revealed that NMN boosting enhanced the levels of mitochondrial DNA-encoded MTCO1 to nuclear DNA-encoded ATP5a ratio (Fig. 5l and Supplementary Fig. 5k), suggesting NMN prompted mito-nuclear protein imbalance in vivo. To determine whether NMN protects mitochondrial homeostasis in terms of mitochondrial superoxide, we quantified the reduction of MitoSox fluorescence with NMN treatment in 5xFAD mice primary astrocytes (Fig. 5m, n). Moreover, the electron microscopic (EM) images showed the effects of NMN on mitochondrial morphology by less damaged mitochondria compared to the vehicle group. We found accumulated mitochondrial vacuolization in the hippocampal tissue of the AD-like 5xFAD mice, while this phenomenon was alleviated with NMN treatment accompanying increased mitophagy (Fig. 5o, p). Finally, the electron transport chain produces a higher adenosine triphosphate (ATP) level after NMN treatment (Fig. 5q), indicating NMN inhibits the collapse of the mitochondria and maintains mitochondrial fitness.

### NMN attenuates Aβ proteotoxicity through the ATF4 pathway

**Fig. 6:**
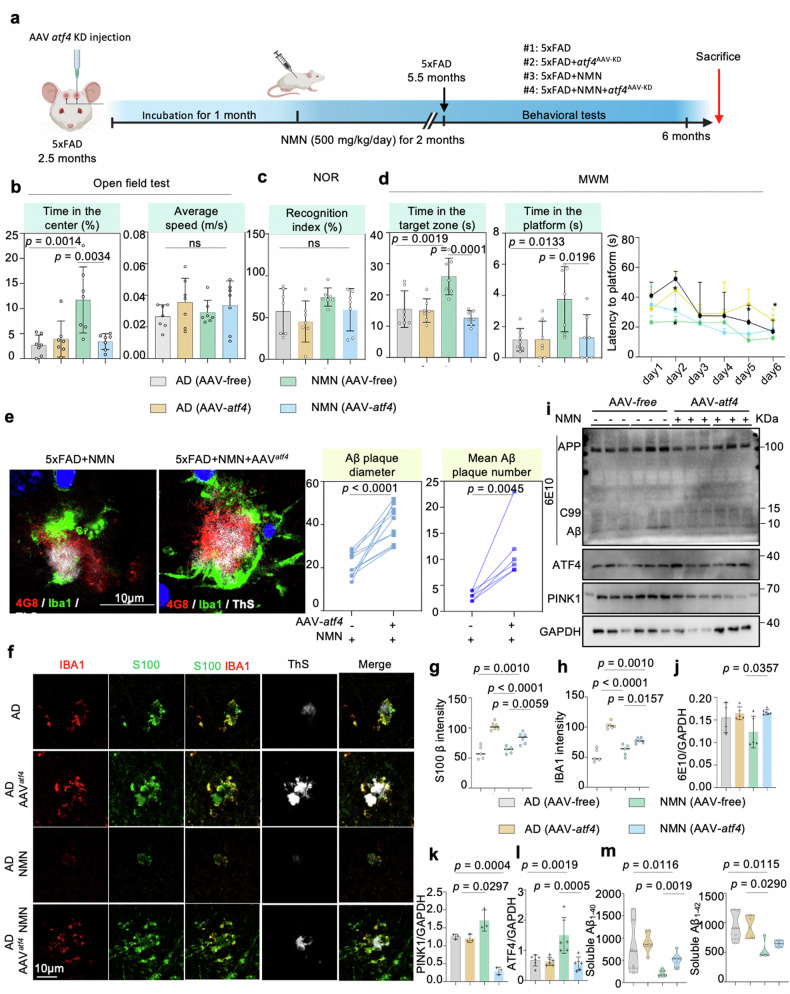
NAD^+^ supplement abrogates Aβ pathological progress and decreases neuroinflammation depending on ATF4-mediated UPR^mt^ in 5xFAD mice. **a** Schematic of NMN injection and behavioral analysis in AAV-*atf4* Knowdown mice. **b**–**d** The behavioral tests analysis of 6-month-old AAV-*atf4* Knowdown mice. Data were analyzed by one-way ANOVA followed by Tukey’s multiple comparisons test for (**b**, **c**), while two-way ANOVA was utilized to assess memory in the MWM test (**d**, *n* = 7 mice per group). **e** Representative immunostained images and quantification of the diameter and number of both 4G8+ (red) and thioflavin S+ (ThS+, white) amyloid plaques, stained with Aβ-positive microglia (IBA1, green) in the hippocampi of AAV-free AD (NMN) and AAV-*atf4* AD (NMN) mice. Scale bar, 10 μm (*n* = 7 mice; two-sided unpaired *t*-test). **f** Representative images showing microglial cells (red) and astrocytes (green) engulfing ThS-immunostained amyloid plaques. Quantification was performed in two independent experiments in at least 10 ROIs. Scale bar, 10 μm. **g**, **h** Corresponding quantification of microglial cells and astrocytes intensity in (**f**) (*n* = 5 mice; two-sided unpaired *t*-test). **i** Effects of NMN on the expression levels of APP and its indicated metabolites, MSR analyses of hippocampal brain tissues samples of 5xFAD mice injected either AAV-free or AAV-*atf4*, following NMN treatment (*n* = 3–6 animals per group). **j**–**l** Corresponding quantification of western blot data in (**i**). Data were analyzed by two-sided one-way ANOVA followed by Tukey’s multiple comparisons test. **m** Soluble and insoluble Aβ_1–40_ and Aβ levels in 5xFAD (NMN) mice injected with AAV-*free* or AAV-*atf4* hippocampal tissues. (*n* = 7 mice; two-sided unpaired *t*-test). All experiments were performed independently with at least three biological replicates with similar results. Data are shown as mean ± s.e.m. The *p*-values are indicated on the graphs. ns not significant.

Notably, NMN has been shown to increase UPR^mt^ signaling in protein levels both in mammals and cells and also reinforce learning and memory capacity in the 5xFAD mice as described above. Thus, to test whether the NMN-mediated memory loss benefit in 5xFAD mice is based on the *atf4* signal, we then assessed the spatial learning and memory of the mice by performing open field tests, NOR, and MWM after 5xFAD mice were injected with *atf4-*AAV-KD (Fig. 6a). NMN improved locomotor activity in the 5xFAD mice as tested using the open field tests at 6 months old, and this benefit was abolished by *atf4* knockdown (Fig. 6b). Next, we checked whether *atf4* knockdown affected the visual recognition memory and learning capacity using the NOR and MWM tasks, respectively. Similarly, as shown in Fig. 6c, d, the memory performance in the 5xFAD mice was enhanced by NMN treatment, and these phenotypes were abrogated by *atf4* knockdown. Notably, during the test period, knockout of *atf4* resulted in significantly increased time to reach the platform even though with NMN administration, decreased preference for the target quarter, and decreased number of platform crossings (Fig. 6d), indicating the NMN attenuates Aβ-mediated memory loss through the ATF4 pathway. Next, to further understand the effect of *atf4* deletion on amyloid pathology within the brain, Thioflavin S (ThS+) and 4G8 (Aβ_17-24_) staining of fixed brain sections were performed, revealed overall more 4G8+ and ThS+ amyloid plaques deposition in 5xFAD (NMN) AAV^*atf-KD*^ mouse brains compared to 5xFAD (NMN) mouse controls (Fig. 6e and Supplementary Fig. 5l, m). In an enlarged view, it was clear that fewer ThS+ Aβ plaques were formed in the NMN-treated 5xFAD mice hippocampus compared to 5xFAD controls in both S100-positive and IBA1-positive glia cells, which was further confirmed by quantification; however, these improvements were eliminated by *atf4* knockdown (Fig. 6f–h). We next examined Aβ levels by western blot. In contrast to the levels of 6E10 in the NMN-treated group were lower than those in 5xFAD mice, a significantly higher 6E10 level was found at the *atf4* deficiency background (Fig. 6i–l). Finally, soluble levels of Aβ_1–40_ and Aβ_1–42_ diminished after NMN treatment, which was also required for *atf4* expression (Fig. 6m). As two main mechanisms of mitochondrial quality control, some researchers pointed out that the supplemented with mitophagy inducer didn’t change UPR^mt^ expression while inhibiting UPR^mt^ would abolish the protective effect of mitophagy [33]. Thus, we next confirmed whether NAD^+^-UPR^mt^ signaling regulates mitophagy in AD. Both of Aβ cell and mouse models displayed that the NMN-induced mitophagy level was reversed by ATF4 knockdown (Fig. 6i and Supplementary Fig. 4e).

### Omics signatures of the hippocampus with NMN in 5xFAD mice

**Fig. 7:**
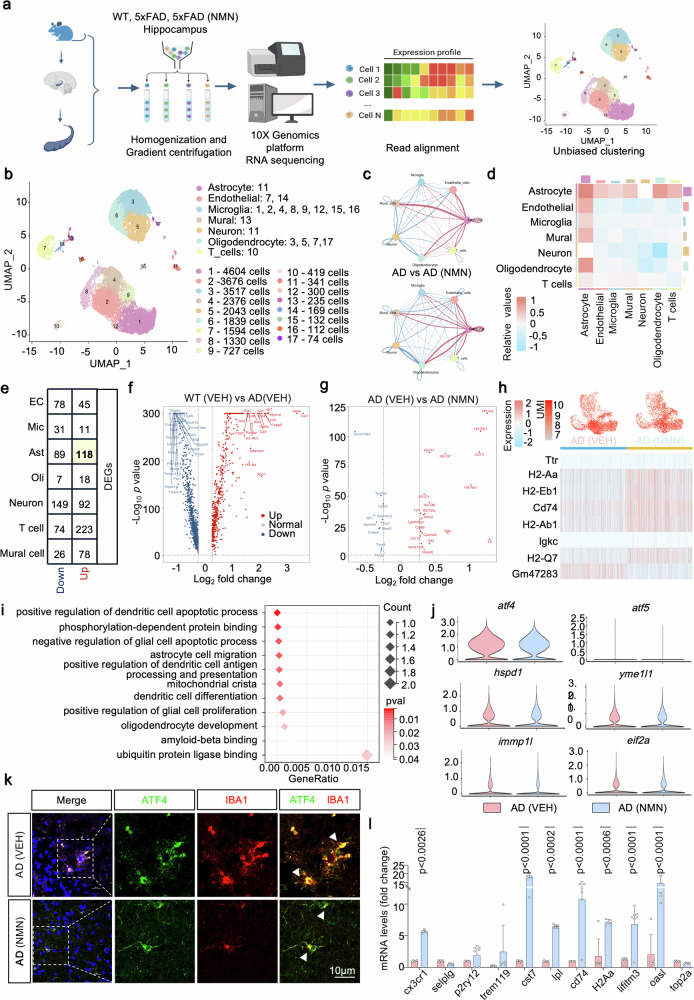
scRNA-seq transcriptional signatures in microglia cells. Brain hippocampal tissues from 6-month-old WT, 5xFAD, and 5xFAD treated with NMN (*n* = 2 per genotype) were subjected to this scRNA-seq. **a** Diagram of snRNA-seq workflow. **b** Two-dimensional uniform manifold approximation and projection (UMAP) plot showing 17 distinguished clusters of single-cell events captured in scRNA-seq. **c**, **d** Interaction number network plot (**c**, top), interaction strength network plot (**c**, bottom), and heat map (**d**) between different cell types in 5xFAD versus 5xFAD treated with NMN. **e** Number of up and downregulated different expression genes (DEGs) in different cell types. **f**, **g** Volcano plots illustrating upregulated or downregulated genes in the WT compared to 5xFAD (**f**) and 5xFAD versus 5xFAD treated with NMN (**g**) in microglia cells. **h** UMI feature plot and heat map of average gene expression of top DEGs in the microglia cluster from 5xFAD and 5xFAD+NMN mice. **i** Functional annotation of certain microglia clusters using GO pathways enriched for their signature genes (mitochondrial-related genes). **j** Violin plots showing average UPR^mt^-related gene-expression across microglia cells. **k** Confocal images of double immunostained with ATF4 (green) and IBA1 (red). The insert is a higher magnification of the boxed area showing the decreased level of ATF4 and upregulated level of IBA1. Arrowheads point to a colocalization of ATF4 and microglia. Scale bar, 10 μm. **l** Transcript levels of marker genes for microglia cluster identification in the hippocampal groups of 5xFAD and 5xFAD+NMN mice (*n* = 6 mice; two-way ANOVA). All experiments were performed independently with at least three biological replicates. Data are shown as mean ± s.e.m. The *P*-values are indicated on the graphs. ns not significant.

Next, we asked in which cell-type NMN-ATF4 functions in the protection of AD. Single-cell RNA sequencing offers an alternative method to tour the cellular heterogeneity of the brain via screening tens of thousands of individual cells [34]. Here, we profiled a unique cellular-level view of transcriptional alterations associated with NMN boosting in AD mouse backgrounds and revealed the cell-type-specific patterns of cellular subpopulations (Fig. 7a). We collected tissue from the hippocampus, a region of the brain that has a vital role in traits that are affected by AD. To subdivide the major cell types in the hippocampus, we established and annotated the primary cell types of the brain by interrogating the expression patterns of known marker genes. In total, unsupervised clustering revealed a total of 7 distinct clusters across all mice identified, including astrocytes, endothelial cells, microglia, mural cells, neurons, oligodendrocytes, and T cells (Fig. 7b). We then adopt CellChat, a tool that can quantitatively infer and analyze cell-cell communication networks from scRNA-seq data [35]. CellChat analysis of these cell subpopulations displayed that the number and strength of signaling interactions between astrocytes and other cell types increased under the NMN background (Fig. 7c, d). Moreover, we found that astrocyte, endothelial cell, and neuron clusters had the most significant DEGs, especially the astrocytes (Fig. 7e).

In the microglia clusters from 5xFAD mice, the volcano plot depicts upregulated and downregulated genes in WT vs. 5xFAD and 5xFAD-NMN-treated compared to vehicle-treated mice, respectively (Fig. 7f, g). We observed an upregulation of genes including *h2-aa, h2-eb1, cd74, h2-ab1, Igkc*, and *h2-q7* (Fig. 7h). Metabolism and protein gene ontology (GO) analyses revealed common perturbed pathways in NMN-cured 5xFAD mice microglia clusters, including protein ubiquitination, protein folding, lysosomal membrane et al. (Fig. 7i). Importantly, multiple genes previously identified in the UPR^mt^ pathway (for example, *atf4*, *atf5*, *chop*, *hspd1*, *hspd9*, and *eif2a*) were comparable in microglia subpopulations between 5xFAD with and without the NMN regimen (Fig. 7j). To validate these findings further, we performed immunostaining for ATF4 and microglia marker IBA1 to verify and found the NMN treatment did not reveal significant differences in the levels of ATF4 in the microglia region (Fig. 7k). Together, these findings indicate that NAD^+^ boosting with NMN is unable to result in transcriptional changes of genes and proteins involved in UPR^mt^ responses within the microglia clusters. Additionally, transcriptional analysis indicated that NMN administration stimulated the transcriptional expression levels of the microglia marker genes, including *cx3cr1*, *cst7*, *lpl, and cd74* et al., suggesting the microglia expanded by the NMN (Fig. 7l).

Then, the microglia cluster was rescaled and re-clustered, revealing five distinct subclusters, including ARM (activated response microglia), CPM (cycling and proliferating microglia), HM (Homeostatic Microglia), IRM (interferon response microglia), and TRM (transiting response microglia) (Supplementary Fig. 6a, b), which were annotated based on differential expression of known cell cluster-type-specific marker genes (Supplementary Fig. 6c). In the SCENIC workflow, co-expression modules between transcription factor (TFs) and candidate target genes are first inferred using GENIE3 or GRNBoost [36]. We utilized the algorithm to score the activity of each regulon in the individual cell of the microglia subcluster and found that the genes in Module 1 (such as *creb5*, *atf3*, *fosb*, *egr1*, and *stat3* et al.) were significantly upregulated with NMN treatment, mainly in the HM, IRM, and CPM subclusters (Supplementary Fig. 6d). Further differentially expressed genes (DEG) analysis between these five subclusters revealed that the top-upregulated genes in the microglia subcluster were associated with immune-related antigen presentation responses and interferon-induced pathways (Supplementary Fig. 6e). We next examined the representation of UPR^mt^ and mitophagy pathway targets in each microglia cluster. Notably, NMN appeared to not affect the expressions of *atf4*, *atf5*, *chop*, *hspd1*, *hspd9*, and *eif2a* in all subclusters, as well as *fkbp8* and *bnip3* (Supplementary Fig. 6f, g). Thus, NMN administration in the 5xFAD had nearly no impact on the MSR induction in the microglia domain.

### ATF4-mediated MSR signatures can be enhanced by NMN in astrocytes

**Fig. 8:**
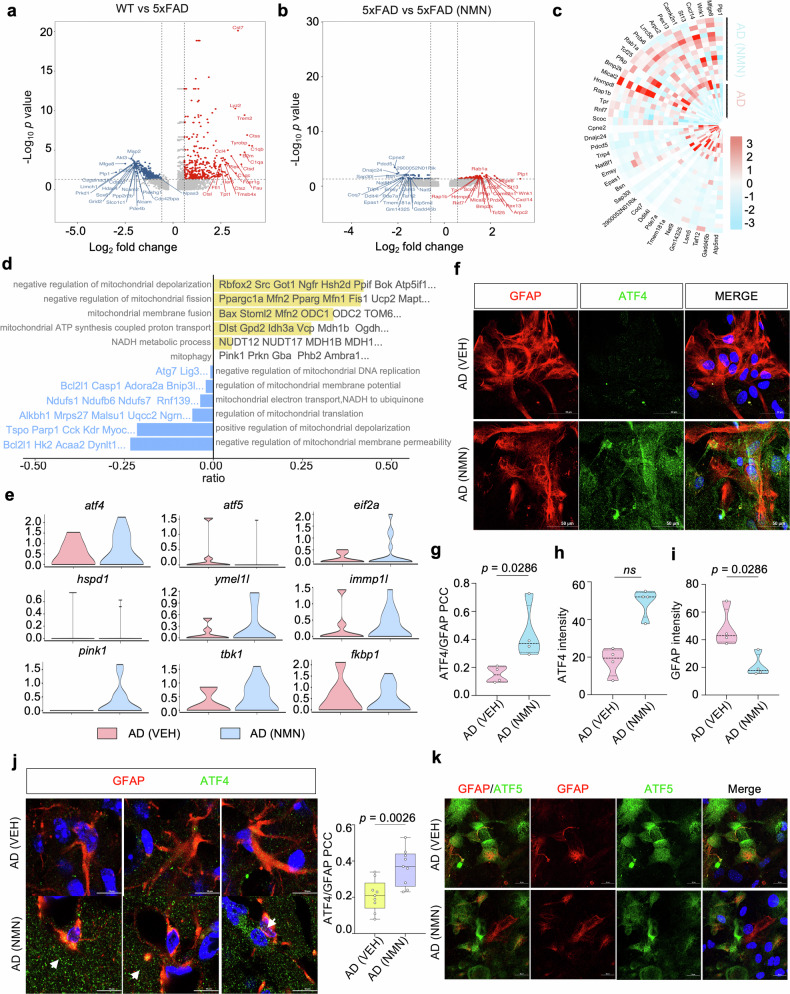
ATF4-mediated MSR signatures can be enhanced by NMN in astrocytes. **a**, **b** Volcano plots illustrating significant DEGs in the WT (VEH) compared to 5xFAD (VEH) and 5xFAD (VEH) versus 5xFAD (NMN) in astrocytes. **c** Heat map revealing the expression of DEGs in astrocytes from 5xFAD (VEH) compared to 5xFAD (NMN). **d** GVSA enrichment analyses for mitochondrial-related pathways in the astrocyte populations of 5xFAD and 5xFAD treated with NMN mice. **e** Violin plots indicating the mean and variance difference between 5xFAD and 5xFAD with NMN in the astrocytes clustering for UPR^mt^ and mitophagy-associated genes. **f**–**i** Representative images of 5xFAD cultured primary astrocytes showing ATF4 staining with GFAP+ astrocytes. Scale bar, 50 μm. **j** Immunofluorescence labeling of 5xFAD and NMN-treated mouse brain with ATF4 and GFAP antibody. The arrow indicates GFAP+ astrocytes were high co-labeling with ATF4 in the NMN-treated AD mouse brain. The experiment was repeated twice independently with similar results. Scale bar, 10 μm. **k** Representative images of 5xFAD cultured primary astrocytes were subjected to co-immunostaining for GFAP and ATF5. Scale bar, 20 μm. All experiments were performed independently with at least three biological replicates. Data are shown as mean ± s.e.m. The *p*-values are indicated on the graphs. ns not significant.

The volcano plot displayed the upregulated and downregulated genes in WT vs. 5xFAD and 5xFAD vs. NMN-treated 5xFAD group in the astrocyte clusters, respectively (Fig. 8a, b). We found an upregulation of genes involved in endocytosis and vesicle transport along microtubule (*rab1a*), phagocytosis of apoptotic cells (*mfge8)*, ATP binding activity (*wnk1*, *hsp90, and arpc2*), the predominant component of myelin (*plp1*), calcium ion homeostasis (*Camk2n1*), and an adaptor protein that mediates the association of the heat shock proteins HSP70 and HSP90 (Fig. 8c). Oppositely, genes involved in the regulation of chaperone-mediated protein folding, macromolecule metabolic processes and mitochondrion organization (*pdcd5*), ATP-dependent activity (*dnajc24*), non-sequence-specific DNA binding activity (*sap30l*), signal transduction (*ddit4l*), protein acetylation (*nat9*), and activity in the mitochondrial inner membrane and nucleus (*coq7*) were downregulated (Fig. 8c). GO term enrichment and GSEA analyses revealed common perturbed pathways in NMN-treated astrocyte clusters, including mitochondrial depolarization, ATP synthesis, mitochondrial translation, et al. (Fig. 8d). Importantly, based on the scRNA-seq analysis, multiple genes involved in UPR^mt^ (*atf4*, *eif2a*, *hspa9*, *ymel1l*, and *immp1l*) and mitophagy (*pink1, tbk1, fkbp1*) were obviously upregulated in the 5xFAD with NMN-treated mice (Fig. 8e). To further test whether increased NAD^+^ biosynthesis in astrocytes can induce the MSR, we performed immunostaining for ATF4 and astrocyte marker GFAP in 5xFAD mice primary astrocytes and found the NMN treatment increased the percentage of ATF4 colocalized to the GFAP compared to the vehicle group (Fig. 8f–i). Furthermore, we quantified the colocalization of ATF4 with GFAP by fluorescence in the 5xFAD mice hippocampus and got the induction of the ATF4 profile within astrocytes (Fig. 8j), suggesting that NMN increased the ATF4 profile mainly in astrocytes. Moreover, to characterize the expression pattern of ATF5 in the astrocytes, we used fluorescent colocalization and found that ATF5 is highly expressed in the GFAP-positive cells in the NMN-treated 5xFAD mouse primary astrocytes as well, with low levels in the vehicle-treated group (Fig. 8k and Supplementary Fig. 5n–p). Together, these results showed that NMN changed the ATF4-mediated MSR signatures in the astrocyte unit.

**Fig. 9:**
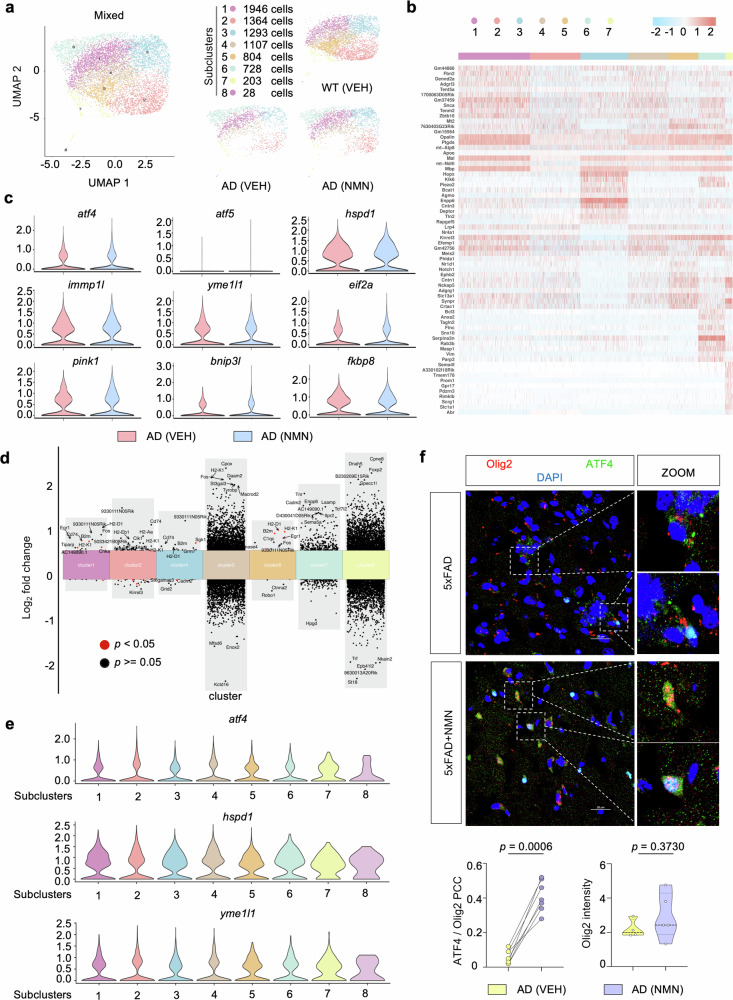
scRNA-seq identifies oligodendrocyte subclusters. **a** Two-dimensional UMAP plots of re-clustered oligodendrocytes showing eight subclusters. Dots are colored based on the scRNA-seq clustering. **b** Heat map showing average gene expression of top DEGs in eight subclusters of oligodendrocytes. **c** Violin plots indicating average gene-expression across oligodendrocytes of mitophagy (*pink1, binp3l, fkbp8*) and UPR^mt^ (*atf4, atf5, hspd1, hspd9, yme1l1, eif2a*) associated genes between 5xFAD versus NMN-treated 5xFAD mice. **d** Multiple volcano plots illustrating significant DEGs in oligodendrocyte subclusters in 5xFAD versus NMN-treated 5xFAD samples. **e** Violin plots showing expression of UPR^mt^ genes in oligodendrocyte subclusters. **f** Representative immunofluorescence images and quantification of Olig2+ oligodendrocytes labeling with ATF4. The enlarged picture indicates Olig2+ oligodendrocyte was high colocalization with ATF4 in NMN-treated mice. The experiment was repeated twice independently. Scale bar, 25 μm (*n* = 3 mice; two-sided unpaired *t*-test). All experiments were performed independently with at least three biological replicates. Data are shown as mean ± s.e.m. The *p*-values are indicated on the graphs. ns not significant.

A recent study showed that oligodendrocyte dysregulation of myelination occurred In AD pathogenesis [37]. Unsupervised clustering revealed a total of 8 distinct oligodendrocyte subclusters across all samples (cluster 1 to cluster 8; Fig. 9a). These subclusters were manually identified based on the expression of known cell-type-specific markers (Fig. 9b). We first performed a detailed analysis of MSR signature changes. Similar to the previous microglia performance, UPR^mt^ and mitophagy pathway targets were comparable in oligodendrocyte populations between 5xFAD with and without NMN (Fig. 9c). We next focused on the DEGs on a subcluster basis, specifically comparing 5xFAD versus 5xFAD plus NMN in datasets obtained from 6-month-old mice (Fig. 9d). Notably, NMN did not impact the expressions of UPR^mt^ indexes such as *atf4*, *atf5*, and *yme1l1* in all oligodendrocyte subclusters (Fig. 9e). To validate these results in vivo, we stained ATF4 with the oligodendrocyte marker olig2 and found that the ATF4 signal and its colocalization with olig2 were detected mild more apparently after NMN supplementation, but without significant (Fig. 9f), suggesting that ATF4 activation is not involved in the oligodendrocyte cluster.

## Discussion

Proteotoxic stress in AD is associated with mitochondrial dysfunction, and impaired mitophagy activity has been considered one of the major hallmarks of this disease [15]. Although there’s an extremely high prevalence in the aging population [38], it is still unknown how MSR evolved, and whether it contributes to AD is poorly understood. Our recent work first demonstrated that during the aging process, plasma UPR^mt^-mitophagy leads to MSR dyshomeostasis in AD subjects. Activation of the NAD^+^-MSR pathway is beneficial for AD-related cross-species models. Of note, several studies indicated that NAD^+^ boosters (mainly NR) result in novel approaches for the treatment of AD and could potentially be translated into the clinic to improve AD patients’ phenotypes [17], but it was unclear how NMN relates to MSR-dependent pathological hallmarks of AD brain dysfunction. Here we provide evidence for a direct interaction between NMN and MSR signature in vitro and in vivo, demonstrating that NMN supplementation ameliorates mitochondrial proteotoxicity, sustains mitochondrial homeostasis, decreases hippocampal synaptic disruption, and decreases neuronal loss as well as brain atrophy. Remarkably, omics features of the hippocampus with NMN in 5xFAD mice showed that NMN leads to transcriptional changes of genes as well as proteins involved in MSR characteristics, principally within the astrocytes rather than the microglia and oligodendrocyte units. Thus, based on our results, we speculate a highly favorable communication between NMN and MSR against amyloid-β aggregation and tau pathology. These findings imply that enhancing mitochondrial function and proteostasis may lessen the detrimental proteotoxic stress in the context of AD.

In this study, we have elucidated that NMN exerts neuroprotective effects on AD through the ATF4-dependent MSR pathway. The homeostatic effects of NAD^+^ on mitochondria are tightly related to ATF4. Furthermore, using scRNA-seq analysis we suggest that astrocytes may be the main location of action of the NMN-ATF4 pathway. ATF4 belongs to the activating transcription factor (TF) family and its activation is enhanced upon the stimulation of a diverse array of stresses [39], which could regulate the expression of multiple genes that are involved in oxidative stress, differentiation, metabolism, and development et al. [40]. The protein level of ATF4 is upregulated in the brains of AD mouse models and AD patients, indicating its latent role in the pathogenesis of this disease [41, 42]. Due to the potential role of ATF4 in neuronal resilience, it is supposed a vital regulator of learning and memory. Ohno found that ATF4 played an important role in the mouse model for synaptic plasticity and memory improvement [43]. On the contrary, Goswami et al. reported that inhibition of ATF4 ameliorated the endoplasmic reticulum stress-induced neuroinflammation and cognitive Impairment in the AD model [44]. Whatever, these results suggest that ATF4 is a novel potential therapeutic target for AD. Moreover, regarding the potential molecular mechanism on the regulation of ATF4 by NAD^+^, we have summarized the literature we have reviewed in the Supplementary Fig. 7.

Of note, several studies reported that other NAD^+^ precursors such as NR and NAM also improved memory competence, mitochondrial function, and increased fitness in animal models of AD, Parkinson’s disease, and aging [45–48]. This suggests that NAD^+^ boosting can reverse the damaged brain-energy metabolism and possibly mitochondrial dysfunction that is implicated in cognitive decline. Recently, Michael J., et al. reported that reversing an age-dependent decline in NAD^+^ through NMN restored oocyte quality, mitochondria bioenergetics, and functional fertility in aged mice [49]. Deletion of *Nampt* in projection neurons of adult mice leads to motor dysfunction, impaired mitochondrial bioenergetics, neurodegeneration, and death. When treated with NMN, *Nampt* cKO mice mimic the amyotrophic lateral sclerosis (ALS) model, and exhibit reduced motor function deficits, enhanced mitochondrial function, and a prolonged lifespan [50]. Moreover, long-term NMN administration mitigates age-associated physiological decline in mice, adjusts energy metabolism, and enhances mitochondrial oxidative response without any apparent toxicity or deleterious effects [51]. However, little is known about NMN and MSR interconnection in the process of AD pathogenesis. To end this aim, we found here that NMN has a dramatic effect on MSR, mitochondrial homeostasis, and behavior deficits in AD mice.

NAD^+^ is a core metabolite involved in cellular bioenergetics, genomic consistency, mitochondrial sustainability, adaptive stress resilience, and cell survival [16]. Moreover, NAD^+^ augmentation enhances neuronal survival and lifts cognitive function in premature aging models. The potential mechanism is likely related to low DNA damage and promote PARP1 activity. Additionally, increased SIRT1 and PGC-1a activities were observed after NAD^+^ boosting, when combined with enhanced DNA repair, improved nuclear-to-mitochondrial communication [47], indicating a decline in NAD^+^ contents contributes to the age-related recession of mitochondrial biogenesis via impaired SIRT1-PGC-1a signaling [52]. Thus, NAD^+^ is critical for the coupling of mitochondrial biogenesis and mitophagy to maintain mitochondrial homeostasis in neurons. We performed the single-cell Metabolism analysis using the VISION algorithm and found that Nicotinate and nicotinamide metabolism pathways were upregulated in astrocytes after supplementation with NMN in 5xFAD mice. On the other hand, mitochondria dysfunction-mediated diseases are one of the most common types of metabolic disorders, which happen at any age or in any organ. Shedding light on this diversity is emerging from recent research showing that cell-specific stress responses are triggered respond to impaired mitochondria [53]. A crucial challenge lies when the disruption of mitochondrial protein quality control contributes to the progression of disease. Hence, the induction of MSR and the activation of quality control pathways initiate protection signals in mitochondria [54]. MSR depends on fusion-fission dynamics that dilute and segregate injured mitochondria. This response involves the induction of genes that carry a series of conserved amino acid response elements in their upstream regulatory region. Then, the element is the binding site of ATFs, different isoforms of which have been linked to the UPR^Er^ or the UPR^mt^ [55]. Currently, we found that NAD^+^ supplementation with NMN improved ATF4-dependent UPR^mt^ -MSR signatures in vivo and in vitro in AD models.

In the context of AD pathology, scRNA-seq showed that NMN improved MSR characteristics, mostly within the astrocyte. Astrocytes are cells that manage metabolic and redox homeostasis, emerging as an important focus of AD research [56]. Increased GFAP immunoreactivity in the vicinity of Aβ plaques was supposed to be a hallmark of reactive astrogliosis in association with AD development [57]. However, advanced transcriptomics studies now imply that more than hundreds of protein targets related to reactive astrocytes are shown to be increased in AD subject brains [58]. Recently, one promising biomarker candidate is the astrocyte-derived α7nAChR, which correlates with Aβ pathology in the brain of individuals with AD [59]. Senescent astrocytes exhibit high levels of β­galactosidase, which accumulate in the brains of people with AD. In vitro, human astrocyte data suggest that Aβ deposition can be a trigger of senescence [60]. One study displayed that the clearance of senescent astrocytes in the tauopathy model diminished tau-mediated neurofibrillary tangle formation, gliosis, and ameliorated cognitive decline [61]. In addition, crosstalk within the glial cell ecosystem (astrocyte–microglia) is essential for neuronal and brain health. Cameron S et al. reported that astrocyte-sourced IL-3, as a key mediator, programs microglia to ameliorate the pathology of Aβ and tau [62]. These data support the potential and promising value of astrocytes to reduce AD-related pathology and provide a plausible node for therapeutic intervention in AD. Notably, one limitation of the current study is the use of N2s cells could be considered not the appropriate or state-of-the-art in vitro model, and similarly, the use of 5xFAD mice as a single in vivo AD genetic model can be questionable. In addition to N2s cells, we also used 5xFAD-derived primary astrocytes and doxycycline-induced tau cell lines, which we hope will increase the diversity of cell models.

## Conclusion

Our work provides evidence that mitochondria have an active role in the pathogenesis of AD, as reducing mitochondrial homeostasis via *atf4* depletion in 5xFAD mice aggravates the hallmarks of the disease; conversely, boosting mitochondrial proteostasis by NMN decreases protein aggregation, restores memory performance, and delays disease progression, ultimately translating to increased healthspan.

## Material and methods

### Analyses of human samples

The cohort consisted of 43 clinically diagnostic AD participants and 46 age-matched healthy controls (HCs) from the First Affiliated Hospital of Wenzhou Medical University between September 2022 and October 2023, according to Alzheimer’s Disease and Related Disorders Association criteria [63]. The study was approved by the institutional Ethics Board Committee of the Wenzhou Medical University First Affiliated Hospital (KY2021-153). All participants provided written informed consent before participating in this study. Plasma samples were obtained through blood centrifugation (3000 × *g* for 10 min) and stored at −80 °C until subsequent analysis. We used the EDTA anticoagulant tube to collect blood and delivered it to centrifugation within 1 h. Plasma ATF4, ATF5, CHOP, PINK1, and Parkin levels were determined using enzyme-linked immunosorbent assay (Jianglai Biotechnology Company, Shanghai, China, http://www.jonln.com). The detailed information of these kits is as follows: No: JL39738 (ATF4), JL39766 (ATF5), JL19693 (CHOP), JL11175 (PINK1), JL11195 (Parkin), respectively.

Continuous variables were analyzed for normality using the Kolmogorov–Smirnov test (K–S test), histogram, and Q–Q plot. Variables were expressed as the mean (SD) and assessed by analysis of covariance (ANCOVA) or Kruskal–Wallis test (K–W test) followed by Bonferroni corrected post hoc comparisons. The associations between plasma MSR signature biomarkers and cognition evaluation scales were tested using Pearson correlations. Finally, the area under the curve (AUC) of receiver operating characteristic (ROC) was applied to evaluate the predictive utility of MSR signature biomarkers alone or combined models, and the difference in the AUC was determined by utilizing DeLong statistics. Statistical analyses were performed using R version 4.3.0. Statistical significance was set at a two-tailed *p* < 0.05.

### Cell culture for mechanistic studies

The N2a, HEK 293 cells were cultured in DMEM containing 10% FBS (Gibco) and 1% Penicillin-Streptomycin (1% PS, Gibco). N2s (N2a cells expressing human Swedish mutant APP695) and HEK 293 cells expressing pTRE3G-cherry-BI promoter-EGFP Tau P301L were gifts from Jia-Hong Lu of Macau University and maintained with DMEM, 10% FBS, 1% PS, and 200 ug/ml G418 (Geneticin elective Antibiotic, Sigma–Aldrich), and grown for at least three generations before subsequent experiments. A high concentration (1000 μg/ml) of G418 was used for selection, and a low concentration (200 μg/ml) was used for maintenance. The expression of EGFP Tau P301L is controlled by adding doxycycline (DOX, 0.2 µmol/ml) to the culture medium before indicated treatments. After designated treatments (MMN 500 µm/ml), cells were subjected to imaging or gathered for Western blot analysis. All cell lines were maintained in the incubator at 37 °C with 5% CO_2_ tested for mycoplasma using mycoprobe following the manufacturer’s instructions.

### Transgenic mice, behavioral, pathological, and mechanistic studies

5xFAD (B6SJL-Tg (APPSwFlLon, PSEN1*M146L*L286V) 6799 Vas/Mmjax) and adult C57BL/6 J wild type (WT) mice were obtained from the Jackson Laboratory (JAX stock no. 000664). All mice were housed in individually ventilated cages (less than five animals) on standardized rodent bedding for at least one week before the start of the experiment. Controlled temperature, humidity, constant 12-h light-dark cycle (lights on at 07:00), and free food/water were available. The 3.5-month-old 5xFAD mice were treated with NMN (500 mg/kg/day) by intraperitoneal (i.p.) injection for two months and subsequently evaluated for behavioral and molecular endpoints. Mice were randomized to 7–8 animals per experimental group according to their body weight. No blinding was used during the testing procedures. As for 5xFAD *atf4* Knowdown mice, 5xFAD amyloid mice were transduced with 1 μl AAV9-*atf4* virus (3.8 × 10^12^ viral genomes per ml) in each hippocampus at 2.5–3 months of age via brain stereotactic injection [64]. All animal care and experiments were approved by the Committee on the Ethics of Animal Experiments of the Wenzhou Medical University (WYYY-AEC-YS-2023-0004).

### Mouse behavioral tests

Several behavioral assays, including Morris Water Maze (MWM), Y maze, novel object recognition (NOR), open field test, and fear conditioning, were used to investigate changes in learning and memory. Mice were adapted to the behavior room, including the background white noise and decoration, for at least 60 min before initiating the assay. Data is recorded using ANYMAZE (v. 7.20) video tracking software.

#### Morris water maze

The MWM test was performed as described previously [65]. The device is a circular white pool (120 cm diameter × 50 cm depth) filled with tap water with nontoxic white paint to opaque, and the temperature is controlled at 22 °C. A 10 cm diameter platform was located 1 cm below the water surface. A dim light was placed within the room, and only distal visual cues were available above each quadrant of the pool to help in the spatial navigation and location of the platform. The experiment lasted 7 days, with 5 trials of experiments conducted each day. On the first day, mice explored clear water to find a platform with a red flag. The platform’s location changed every trial. Mice with successful consecutive findings within 60 s have good vision, while those who can’t are excluded. On the second day, the water became white, and the platform stayed in a constant position. Mice are put in water from four directions and penalized with a 10-second standing if they can’t find the platform in time but are guided to it. On the 7th day, the platform is removed, and mice can explore freely for 60 s.

#### Y maze

Y-maze consists of three arms of equal length (30 cm × 15 cm × 8 cm), each separated by an angle of 120°, used to assess spatial memory and reference memory. We placed mice in the center of the Y maze and allowed them to explore freely for 8 min, while the video system recorded their activities for 8 min [66]. The following indicators were recorded: (1) the total number of entries: the number of times the animal entered the maze arm; (2) alternation: consecutively entering all three arms of the Y maze once (ABC, ACB, BCA, etc.). spontaneous alternation performance (SAP) = [alternation/(the total number of entries-2)] × 100%

#### Fear-conditioning test

Fear conditioning was conducted as described previously [67]. On Day 1, the mice were given 120 s to freely explore the chamber. They were then exposed to a 2 s stimulus tone (5 Hz, 70 dB), which served as the conditional stimulus, followed by a 0.7 mA foot shock as the unconditional stimulus. This lasted for a total of 5 min. On Day 2, the mice underwent a contextual and cued test session. The contextual test session involved allowing the mice to freely explore the chamber for 5 min with no stimuli. After a 3-h rest, the cued test session was performed, and a white plastic insert was placed in the chamber. Followed by auditory stimuli (30 s each, repeated 5 times with 30-s intervals). The freezing time was recorded to evaluate contextual and cued memory.

#### NOR (novel object recognition) [68]

The experiment consists of two stages: a training and a testing period, conducted in a room with four identical squares (25 × 25 × 25 cm^3^). During the training period, two identical objects are placed in the chamber at the same distance from the surroundings. The mice are allowed to explore for 10 min freely. After 1 h, one of the objects was replaced with a significantly different shape. The mice continued to explore for 10 min. The time and frequency of exploration of the new and old objects were recorded. The Recognition Index (RI) = (the number of explorations of the new object/total number of explorations) × 100%.

#### Open field test

The device was a 25 × 25 × 25 cm^3^ chamber. Mice were placed into the behavioral room 3 h in advance; 75% alcohol was used to remove the smell; the test was started after the mice were put in the middle of the arena for 1 min, and the mice were allowed to explore freely for 10 min. All the data of the mice in the arena were recorded, including the total distance, the time spent in the corners and the center, the number of times they entered the central area, and so on.

### Preparation of brain tissues and procedures

Mice were given a lethal anesthetic dose by i.p. injection of 1% pentobarbital (50 mg/kg in saline). For tissues used in Western blot and ELISA, such as the hippocampus and cortex, we perfuse with physiological saline, remove the whole brain, excluding the cerebellum and medulla, and preserve the remaining parts at −80 °C. We perfuse tissues for immunohistochemistry and immunofluorescence with physiological saline and fix the brain using 4% paraformaldehyde (PFA) at 4 °C. The extracted brain is stored at −4 °C for subsequent freezing and paraffin embedding. For paraffin embedding, the brains immersed in 4% PFA at 4 °C overnight are placed into a pre-labeled embedding cassette. After rinsing with running water for 6 h to remove the 4% PFA, the tissue was placed in 75% ethanol at room temperature overnight. The next day, the tissue is sequentially soaked in 85% ethanol for 2 h, 95% ethanol for 1 h (twice), 100% ethanol for 40 min (twice), 100% ethanol: xylene (1:1) for 30 min, and 100% xylene for 20 min (twice). Finally, the tissue is immersed in soft paraffin for 2 h and hard paraffin for 1 h. After completing all these steps, the tissue is embedded, and the paraffin block is allowed to solidify at 4 °C for long-term storage. For frozen embedding, the whole brain was soaked in 20% sucrose and then 30% sucrose for dehydration. After dehydration, a layer of O.C.T. (Sakura) was placed at the bottom of the embedding cassette, and the prepared brain tissue was put into the cassette. The brain tissue was covered with O.C.T., and the cassette was immediately placed in a −80 °C freezer for storage.

### Immunohistochemistry, immunofluorescence

Cells were seeded in a 12-well plate until reaching 80% confluence. After the corresponding treatment, cells were washed with PBS 3 times, fixed with 4% PFA for 15 min, and washed with PBS 3 times, 5 min each time. Then, cells were incubated with 5% BSA and 0.3% Triton X-100 blocking buffer at 37 °C for 30 min and incubated overnight with 1% BSA-diluted primary antibody at 4 °C. After incubation, cells were washed with PBS 3 times, 5 min each time, and incubated with a corresponding fluorescent secondary antibody (Abcam) for 1 h at 37 °C (protected from light). Following the incubation, cells were washed with PBS 3 times (5 min), and mounted with an anti-fluorescent quenching reagent containing DAPI. Tissue paraffin section: 5 µm thick sections were baked in a 65 °C oven for 30 min and immediately placed in xylene for dewaxing, twice for 15 min each time. Then, the sections were immersed in 100% ethanol, 95% ethanol, 80% ethanol, and distilled water for 5 min each, followed by washing with PBS 3 times (5 min). Frozen tissue section: 30 µm thick sections were kept at room temperature for 1 h to thaw, and excess OCT was washed away with PBS. The prepared sections were subjected to antigen retrieval using a boiling sodium citrate solution. After the solution reached room temperature, the sections were washed with PBS for 3 times. The following steps were the same as the cell culture section. Images were taken with a Nikon C2si and A1R-SIM-STORM confocal microscope. Z-stack images of the stained sections were acquired using a confocal microscope and converted into maximum z-stack projection images.

Specific primary antibodies used include: Anti-Tau (phospho S396) antibody (catalog no.ab109390; Abcam), Anti-Tau5 antibody (catalog no.ab80579; Abcam), 4G8 (Purified anti-β-Amyloid, 17–24) antibody (catalog no.800712; Biolegend), anti-human sAPPa (clone 6E10) antibody (catalog no.803014; Biolegend), anti-BACE-1 (clone D10E5) antibody (catalog no.5606; Cell Signaling), APP Antibody(catalog no.AF6084; Affinity), ATF4 antibody (catalog no.DF6008; Affinity), CHOP antibody (catalog no. AF6277; Affinity), HSP70 antibody (catalog no.AF5466; Affinity), ATF5 antibody (catalog no.DF3480; Affinity), PINK1 antibody (catalog no. A7131; ABclonal), Parkin antibody (catalog no. A0968; ABclonal), LC3B (D11) antibody (catalog no. 3868; Cell Signaling), caspase-3 antibody (catalog no.19677-1-AP; protein tech), cytochrome c (A-8) antibody (catalog no.sc-13156; Santa Cruz), LAMP-1(H4A3) antibody (catalog no.sc-20011; Santa Cruz), Tom20(F-10)antibody (catalog no.sc-17764; Santa Cruz), Cathepsin D (D-7) antibody (catalog no.sc-377299; Santa Cruz), Lamp-1 antibody (catalog no. AB2971-25UG; Santa Cruz), NeuN antibody (catalog no.ab177487; Abcam), beta III tubulin antibody (catalog no. ab78078; Abcam), a-tubulin antibody (catalog no.ab289875; Abcam), GFAP antibody(catalog no.sc-33673; Santa Cruz), GFAP antibody (catalog no. DF6040;Affinity), S100 beta antibody (catalog no.ab52642; Abcam), ibal1 antibody (catalog no.ab283319; Abcam), Olig2 antibody (catalog no.ab109186; Abcam). For the intention of Aβ plaques evaluation, tissues were stained with 6E10 antibody (Biolegend) and 4G8 antibody (Biolegend) to recognize Aβ. For thioflavin S staining, blocked paraffin-embedded 5-μm sections were incubated with 0.1% thioflavin S (Sigma, T1892) for 8 min at 37 °C, protected from light, immersed twice in 80% ethanol and once in 95% ethanol for 3 min each. Afterward, it was followed by three washes in ddH_2_0 water and then stained with the other subsequent experimental steps.

### Western blotting

Western blot assay was done as previously described [15]. The protein concentration of each sample from different cell lines and mouse brain tissues was tested using the Bradford protein assay using 1× RIPA buffer (catalog no. P0013B; Beyotime) containing protease (catalog no. ST506-2; Beyotime) and phosphatase inhibitors (catalog no. P1260; Applygen). Brain homogenates were diluted and denatured in loading buffer, sonicated (3 × 5 s), heated to 100 °C for 5–8 min, and loaded (approximately 50 μg of protein) on a Bis-Tris 7.5–12.5% or Tris-Tricine 10–20% gradient gel with a current of 80 V for 120 min. After electrophoresis, gels were transferred to an immobilized PVDF membrane in a Western Blot Transfer Buffer (250 mA, 90 min) on ice. Blocking was performed with 5% milk for 2 h at room temperatures, then washed 3 times. Subsequently, membranes were incubated overnight at 4 °C with the primary antibody in TBST.

Primary antibodies used were Tau (D1M9X) antibody (catalog no.46687S; Cell Signaling), Phospho-Tau (Thr217/Thr534) antibody (catalog no. AF3913;Affinity), Phospho-Tau (Thr231) [Thr548] antibody (catalog no. AF3147;Affinity), Phospho-Tau (Ser202) [Ser519] antibody (catalog no. AF2419; Affinity), Phospho-Tau (Thr181) [Thr498] antibody (catalog no. AF3149;Affinity), anti-APP-CTF (1:1,000,) antibody (catalog no. A8717; Sigma), LONP1 antibody (catalog no.DFDF12119; Affinity), CLPP antibody (catalog no.DF8448; Affinity), YME1L1 antibody (catalog no.DF12024; Affinity), Beclin-1 (D40C5) antibody (catalog no.3495; Cell Signaling), p62 antibody (catalog no.P0067; Sigma), BNIP3L/Nix antibody (catalog no.12396; Affinity), FUNDC1 antibody (catalog no. AF0002; Affinity), OPTN antibody (catalog no.A1845; ABclonal), Bcl-2 (D17C4) antibody (catalog no.3498; Cell Signaling),RIPK1 antibody (catalog no.ab32503; Affinity), RIPK3 antibody (catalog no.DF10141; Affinity), MLKL antibody (catalog no.DF7412; Affinity), caspase-3 antibody (catalog no.19677-1-AP; protein tech), ATP5a1 antibody (catalog no.DF3806; Affinity), MTCO1 antibody (catalog no.DF8920; Affinity). After membranes were washed 3 times, they were then probed with the respective secondary horseradish peroxidase (HRP)-labeled antibodies. Secondary antibodies, including goat anti-mouse IgG (H + L) HRP (IgG; catalog no. GAM007) and goat anti-rabbit IgG (H + L) HRP (catalog no. GAR007), were obtained from MULTISCIENCES. Densitometric values of single protein bands (amyloid-β, APP, BACE-1, et al.) or fragments or aggregates were analyzed with the software package Image Lab Software (Bio-Rad v 6.1) and normalized to GAPDH or β-actin. Gamma adjustment was applied to reduce the dark background when necessary. All data is analyzed using Excel and Prism (version 9.3.1).

### Enzyme-linked immunosorbent assay (ELISA) for Aβ_1–40_ and Aβ_1–42_

For mice, measurement of Aβ was done using ELISA with minor modifications [15]. The hippocampal and cortical preparations of mice were collected and used to measure soluble Aβ. The resulting insoluble pellet was homogenized with 70% formic acid. Samples were centrifuged at 100,000 × *g* for 20 min at 4 °C, and supernatants were collected. Samples were neutralized in 1 M Tris buffer (pH 11, 1:20 ratio dilution) and diluted in blocking buffer before loading on ELISA. Aβ in the RIPA fraction was regarded as the detergent-soluble fraction, while Aβ in the neutralized formic acid fraction was considered the detergent-insoluble fraction. We used the human Amyloid β ELISA kit from the R&D system to identify soluble and insoluble Aβ_1-40_ and Aβ_1-42_ levels in mice (Aβ_1-40_: catalog no.DAB140B, Aβ_1-42_: catalog no.DAB142).

### RNA isolation and real-time quantitative PCR (qPCR) analysis

An appropriate amount of Trizol was added to an enzyme-free EP tube containing homogenized brain tissue and cells to extract RNA from brain tissue and cells. The mixture was left to stand for 15 min and vigorously pipetted to ensure cell fragments were well mixed. A 1:5 ratio of Trizol to chloroform (1 ml Trizol with 200 μl chloroform) was added, and it was shaken up and down for 10 s at room temperature for 10 min. Then, it was centrifuged at 12,000 rpm for 20 min at 4 °C, resulting in three layers in the EP tube. The supernatant layer, containing the RNA, was transferred to a new EP tube, and an equal volume of isopropanol was added. The tube was shaken up and down 10 times and left for 20 min. The tube was then centrifuged at 12,000 rpm for 20 min again, and a white precipitate at the bottom indicated the presence of RNA. Removed the supernatant, and 1 ml of 75% ethanol was added, shaking the tube to allow the RNA to float. After standing still for 5 min, the tube was centrifuged at 12,000 rpm for 10 min at 4 °C. After discarding the supernatant, the EP tube was inverted on filter paper to allow the alcohol to dry up. The RNA was then dissolved in an appropriate amount of DEPC water. The RNA was quantified using a Nanodrop, and cDNA was synthesized using TOROIVD®qRT Master Mix reagent (catalog no.RTQ-100). Real-time PCR was performed using TOROGreen®qPCR Master Mix (catalog no.QST-100), and all quantitative calculations were performed using the ΔΔCT method. Supplementary Table 1 lists all primers used in this experiment.

### Detection of mitochondrial parameters

#### Electron microscope

The ultrastructure of the mitochondria and nucleus were visualized and imaged with the electron microscope using the method previously reported [15]. In brief, after killing the mice with the standard procedures and a quick collection of the designated brain tissues. Veh- and NMN-treated mouse hippocampal tissues (1 mm in width) were fixed with an appropriate aldehyde fixative (2% paraformaldehyde-2.5% glutaraldehyde electron microscope fixative). After washing with 0.1 M phosphate buffer (pH 7.2) three times, the tissues were post-fixed in 1% osmic acid at 4 °C for 2 h. The samples were gradient dehydrated in a series of 70–100% ethanol baths. Subsequently, the samples were placed in a 50:50 mixture of acetone and embedding media (Embed 812, Electron Microscopy Sciences) for a minimum of overnight. The following day, specimens are placed in a fresh change of pure resin for 4 h, then embedded in another fresh change of 100% resin and put in a 60 °C oven for 12–18 h for polymerization. After the semi-thin (1–2 microns) section was used for positioning with glass knives, the ultra-thin (70–90 nm) section using a diamond knife was made and collected for microstructure analysis, followed by counter-staining with 3% uranyl acetate and 2.7% lead citrate. The samples were then observed with an HT7800 transmission electron microscope. We used Fiji software to analyze mitochondrial ultrastructure and measure mitochondrial diameter, area, and number.

#### MitoSOX and Mitotracker stainings

Diluted the pre-prepared 5 mmol/L MitoSOX™ solution (Invitrogen™, catalog no.M36008) at a ratio of 1:1000, and add it to pre-warmed PBS-washed cell culture dishes [69]. Cells were incubated at 37 °C for 10 min, protected from light. For staining with Mitotracker™ Deep Red FM (Invitrogen™, catalog no.M22426), diluted at a ratio of 1:5000–1:50000 and added to the cell culture dishes [70]. Incubate at 37 °C for 30 min. After being washed with pre-warmed PBS three times, it was stained with DRAQ5 (Thermo Scientific, catalog no.62254) and immediately observed under a microscope.

### Magnetic resonance imaging (MRI) studies

High-resolution T2-weighted images and diffusion tensor imaging (DTI) were obtained using a 9.4 T BioSpec 117/16 USR scanner (Bruker BioSpin, Germany), operated with ParaVision® 6.0 (Bruker BioSpin), using orthogonal 1H volume coils (for mouse head imaging, ≥23 mm inner diameter) [71]. After all acquired images were graphically aligned with Slicer (v5.2.2), cortical thickness was measured, and subsequently, ROIs were circled using Fiji to calculate the corresponding ventricular volumes.

### Isolation and culture of primary astrocytes

Mice within 24 h of birth were sterilized with 75% ethanol, and whole brains were removed using autoclave instruments and transferred to dissecting dishes containing pre-cooled HBSS (Gibco). The olfactory bulb and cerebellum were removed under a microscope, the meninges in the cortex were carefully peeled off, and the separated hemispheres were transferred to a new HBSS, operating on ice throughout. The brain hemispheres were immediately cut up and transferred to a new centrifuge tube. 0.25% trypsin was added and placed in a 37 °C cell incubator to digest the tissue for 30 min, with the tube being flicked every 10 min to aid better digestion. The digestion was terminated, filtered using a 70 µm sterile strainer, followed by centrifugation, and the obtained cells were collected and inoculated in culture flasks using DMEM (Gibco) and incubated in a cell incubator (37 °C, 5% CO_2_). The culture medium was changed on the 2nd day and every 3 days after that until the astrocytes were confluent on the 7th–8th day. Then, the microglial cells were removed by a thermostatic shaker at 37 °C for 180 r/min for 30 min, and the medium was changed. Subsequently, the oligodendrocyte precursor cells were removed at a condition of 37 °C for 240 r/min for 6 h, and the rest of the cells were collected and passaged to obtain pure astrocytes.

### Single-cell RNA sequencing

Brain hippocampus cell populations were prepared from WT and 5xFAD with and without NMN administration. For droplet-based single-cell RNA sequencing (scRNA-seq), libraries were prepared using the Chromium Single Cell 3′ Reagent Kits v.3 (No. PN-1000075) according to the manufacturer’s protocol (10x Genomics). The generated snRNA-seq libraries were sequenced using NextSeq 500/550 High Output v3 kits (150 cycles). Gene counts were obtained by aligning reads to the hg38 genome using CellRanger software (v.2.0.0) (10x Genomics). The procedures for the snRNA-seq are as follows: (1) GEM Generation & Barcoding: prepare the master mix and transfer GEMs; (2) Post GEM-RT Cleanup & cDNA Amplification; (3) 3ʹ Gene-expression Library construction; (4) Post Library Construction QC. Markers genes of cell-type clusters (e.g., neurons) and microglia cell-state signatures were obtained from previously published studies [72].

### Quality control and normalization

The initial dataset contained 9160~9723 cells. The whole dataset was projected onto the two-dimensional space using UMAP on the top 7 main targets as an initial reference. For each cell, the following quality measures were quantified: (1) the number of genes for which at least one read was mapped; (2) the total number of counts; (3) the percentage of counts mapping to the top 50 genes; and (4) the percentage of reads mapped to mitochondrial genes (which may be used to approximate the relative amount of endogenous RNA and is commonly used as a measure of cell quality). According to these observations and subsequent scatter-plot analyses, cells with fewer than 200 detected genes and cells with an abnormally high ratio of counts mapping to mitochondrial genes were removed. After excluding doublet, multicellular, and apoptotic cells, the final number of cells obtained ranged from 7669 to 8068. The average number of UMI per cell ranged from 9991 to 10860, and the average number of genes per cell ranged from 3040 to 3288. The average mitochondrial UMI percentage per cell ranged from 0.0320 to 0.0450. Principal component analysis (PCA) and uniform manifold approximation (UMAP) were performed.

### Clustering and finding markers

Differential gene-expression analysis was performed by the FindMarkers function of the Seurat package for identifying markers for different clusters, using the presto method by default [73]. The dimensionality reduction results based on PCA are visualized through UMAP for single-cell clustering. The clustering algorithm utilized is the Shared Nearest Neighbor (SNN) algorithm, which ultimately obtains the optimal cell grouping. Three differentially expressed gene groups are identified, with 9, 485, and 595 differentially expressed genes detected. After dimensionality reduction and clustering, 17 cell clusters were identified. The presto method analyzed differential expression between a specified cell cluster and all others.

### Differential gene-expression analysis

snRNA-seq-based DEGs were assessed using two tests. First, a cell-level analysis was performed using the Wilcoxon-rank-sum test and FDR multiple-testing correction. Second, a Poisson mixed model accounting for the individual of origin for nuclei and unwanted sources of variability was performed using the R packages lme4 and RUV-seq, respectively. The consistency of DEGs detected using the cell-level analysis model was assessed by comparing the directionality and rank of DEGs. Consistency in directionality for all cell types was quantized by counting the fraction of the top 1000 DEGs (ranked by FDR scores) detected in cell-level analysis that showed consistent direction in the mixed model. We further tested whether the differential *P* value and z-score ranks corresponded to genes detected as upregulated or downregulated in the cell-level analysis when computed using the mixed model. For analyses involving DEG levels, only genes that were significantly approved by both models using the criteria FDR-corrected *p* < 0.01 in a two-sided Wilcoxon-rank-sum test, absolute log_2_ > 0.25, and FDR-corrected *p* < 0.05 in a Poisson mixed model were considered. The differential analysis was performed by fitting a linear model using the R package limma. Moreover, enrichment analyses were performed using the R package ClusterProfiler and the *p*-value-ranked gene lists as input.

### Statistical analysis

All quantified data show an average of samples, and no statistical methods were used to predetermine sample sizes. Data are presented as mean ± s.e.m. unless otherwise specified. Two-tailed unpaired *t*-tests were used for comparisons between the two groups. Group differences were analyzed with a one-way analysis of variance (ANOVA) followed by Šidák’s multiple comparisons test or a two-way ANOVA followed by Tukey’s multiple comparisons test for various groups. Prism 8.0 (GraphPad Software) was used for the statistical analysis. *P*-values < 0.05 were considered statistically significant.

## Supplementary information

Supplementary documents

Original Data

## Data Availability

The main data supporting the findings of this study are available within the article and its Supplementary Information. The custom code for scRNA-seq is available from the corresponding author on reasonable request.
