# Supplementary material for: NAD+-boosting agent nicotinamide mononucleotide potently improves mitochondria stress response in Alzheimer’s disease via ATF4-dependent mitochondrial UPR
Source: Cell Death Dis. 2024 Oct 11;15(10):744. doi: 10.1038/s41419-024-07062-1 (PMC11470026; doi:10.1038/s41419-024-07062-1)
Supplement: Supplementary file 1 — Supplementary documents [file 41419_2024_7062_MOESM1_ESM.pdf]

Supplementary documents

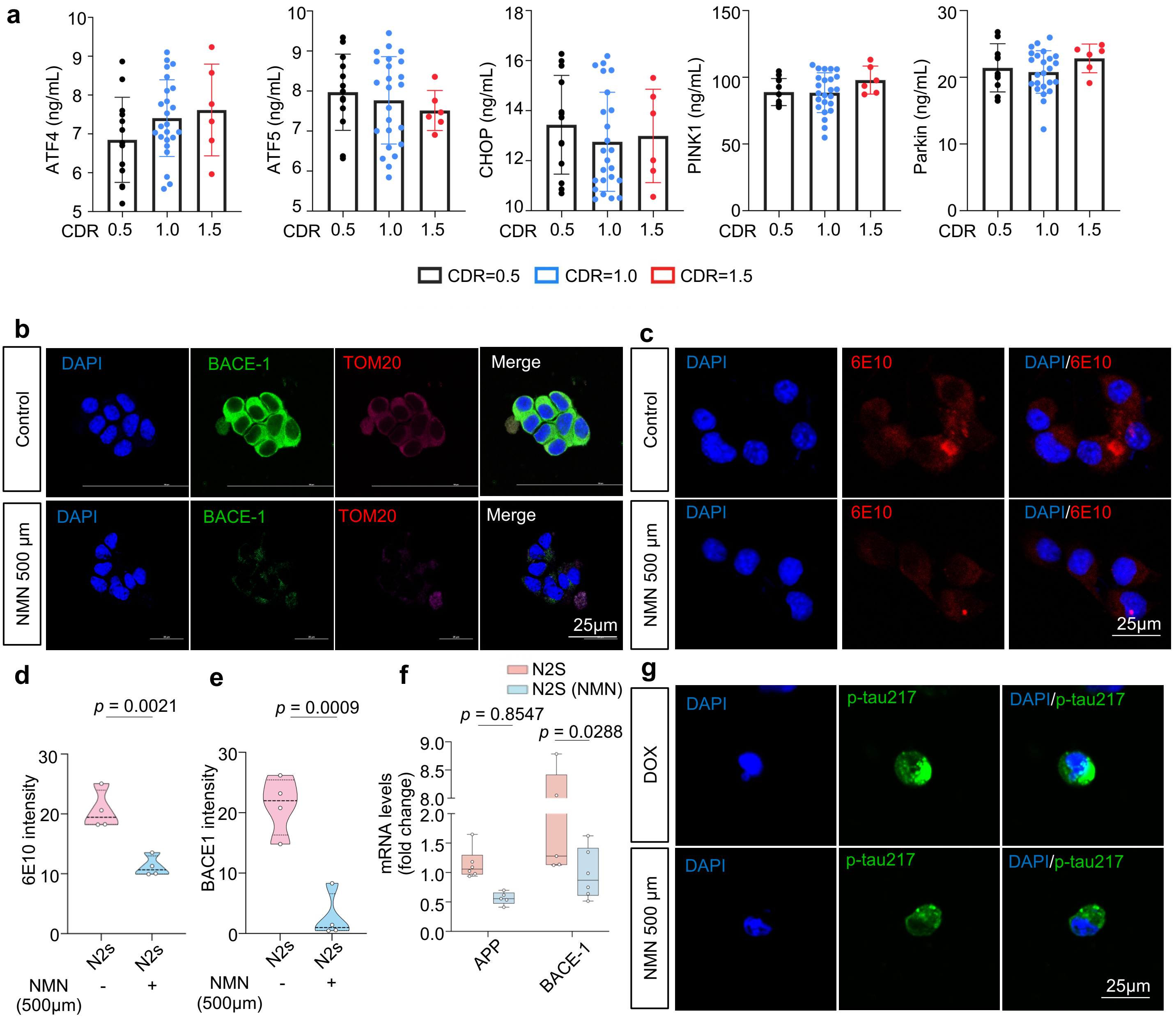

Supple Figure 1

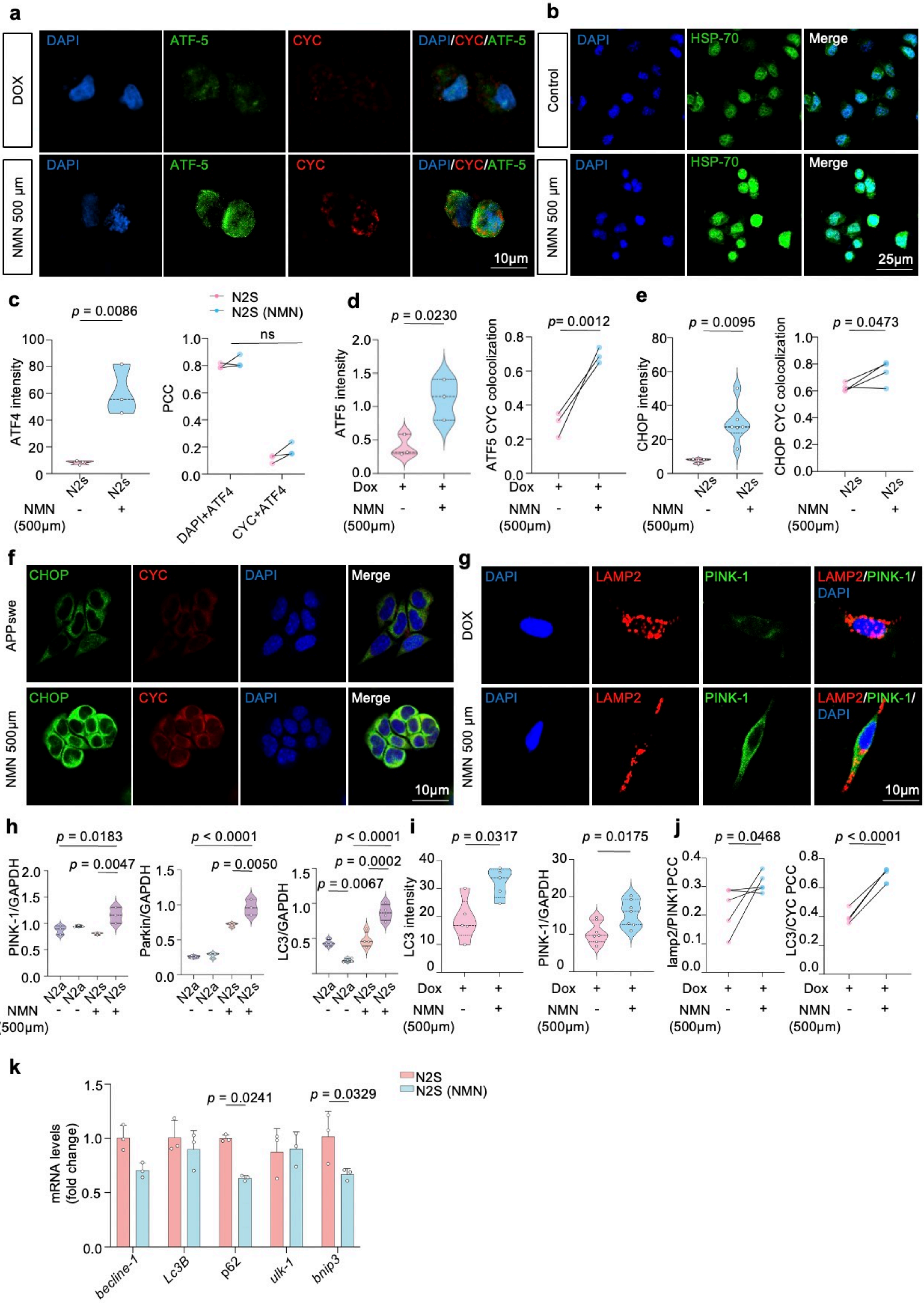
a
DOX
NMN 500 µm
DAPI
ATF-5
CYC
DAPI/CYC/ATF-5
10µm
b
Control
NMN 500 µm
DAPI
HSP-70
Merge
25µm
c
p = 0.0086
ATF4 intensity
N2s
NMN (500µm)
N2S
N2S (NMN)
ns
PCC
DAPI+ATF4
CYC+ATF4
d
p = 0.0230
ATF5 intensity
p= 0.0012
ATF5 CYC colocolization
Dox
NMN (500µm)
e
p = 0.0095
CHOP intensity
p = 0.0473
CHOP CYC colocolization
f
APPswe
NMN 500µm
CHOP
CYC
DAPI
Merge
10µm
g
DOX
NMN 500 µm
DAPI
LAMP2
PINK-1
LAMP2/PINK-1/DAPI
10µm
h
p = 0.0183
p = 0.0047
PINK-1/GAPDH
p < 0.0001
p = 0.0050
Parkin/GAPDH
p < 0.0001
p = 0.0002
p = 0.0067
LC3/GAPDH
N2a
N2s
NMN (500µm)
i
p = 0.0317
LC3 intensity
p = 0.0175
PINK-1/GAPDH
j
p = 0.0468
lamp2/PINK1PCC
p < 0.0001
LC3/CYC PCC
k
N2S
N2S (NMN)
mRNA levels (fold change)
p = 0.0241
p = 0.0329
becline-1
Lc3B
p62
ulk-1
bnip3

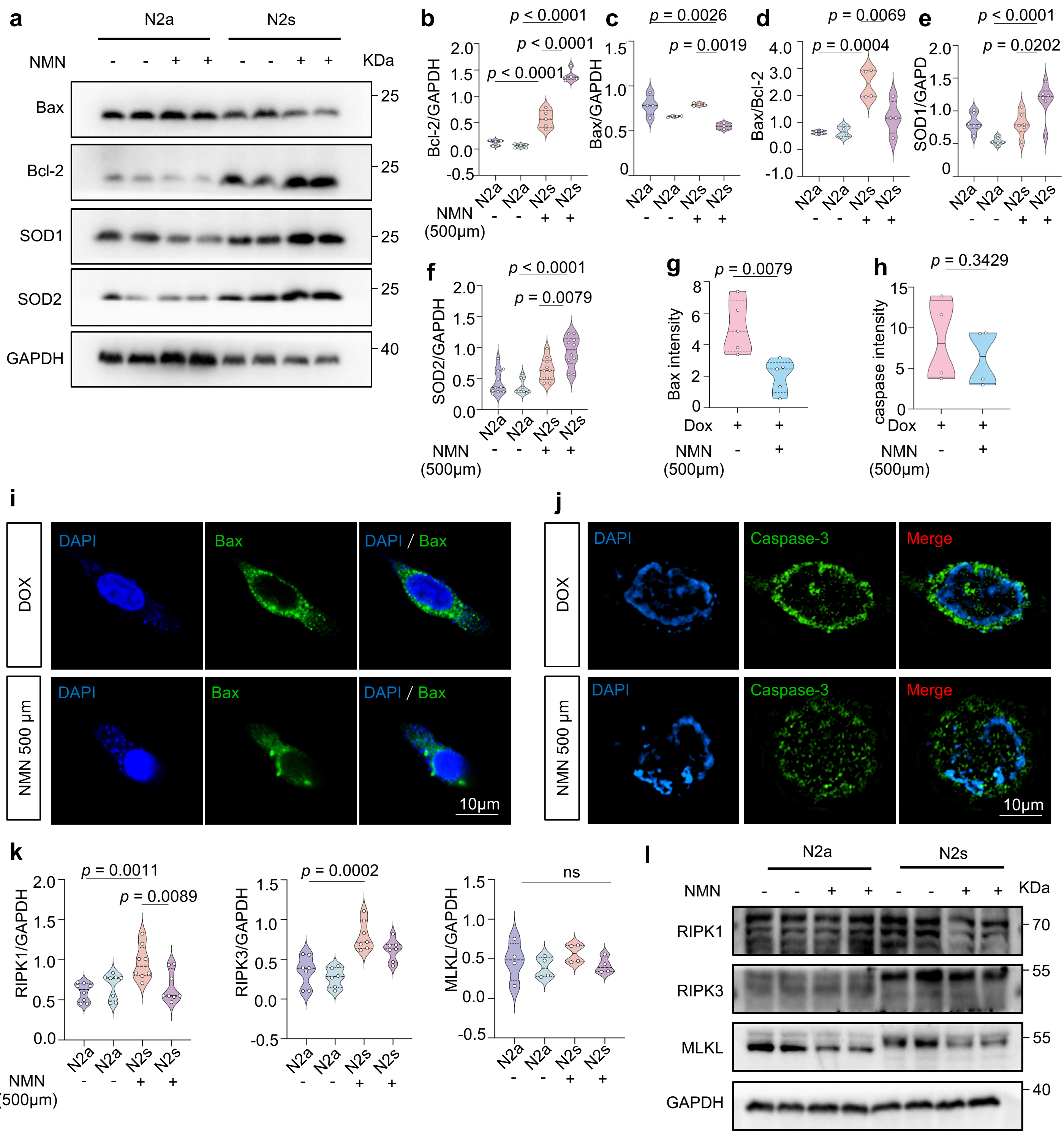

Supple Figure 3

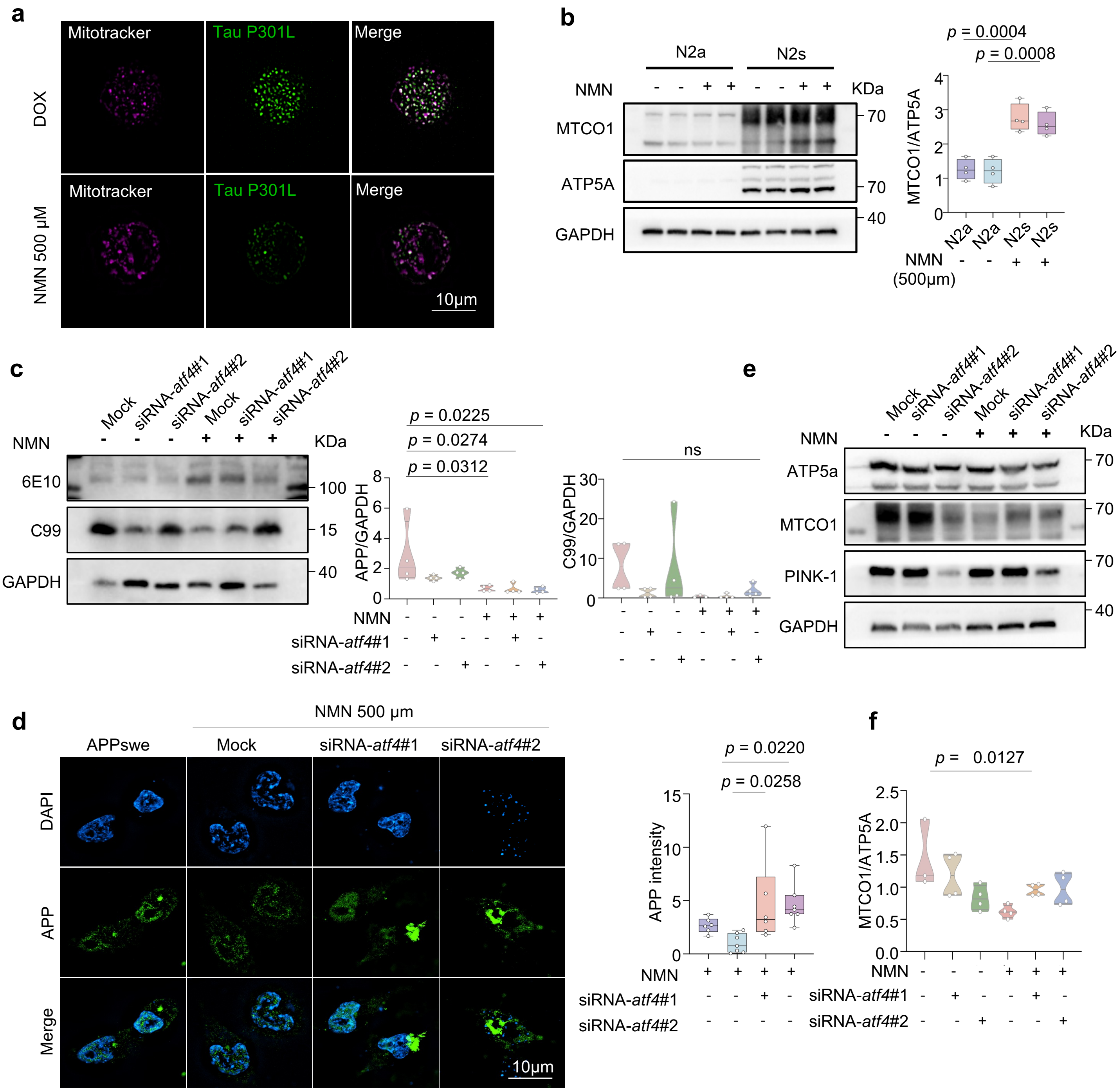

Supple Figure 4

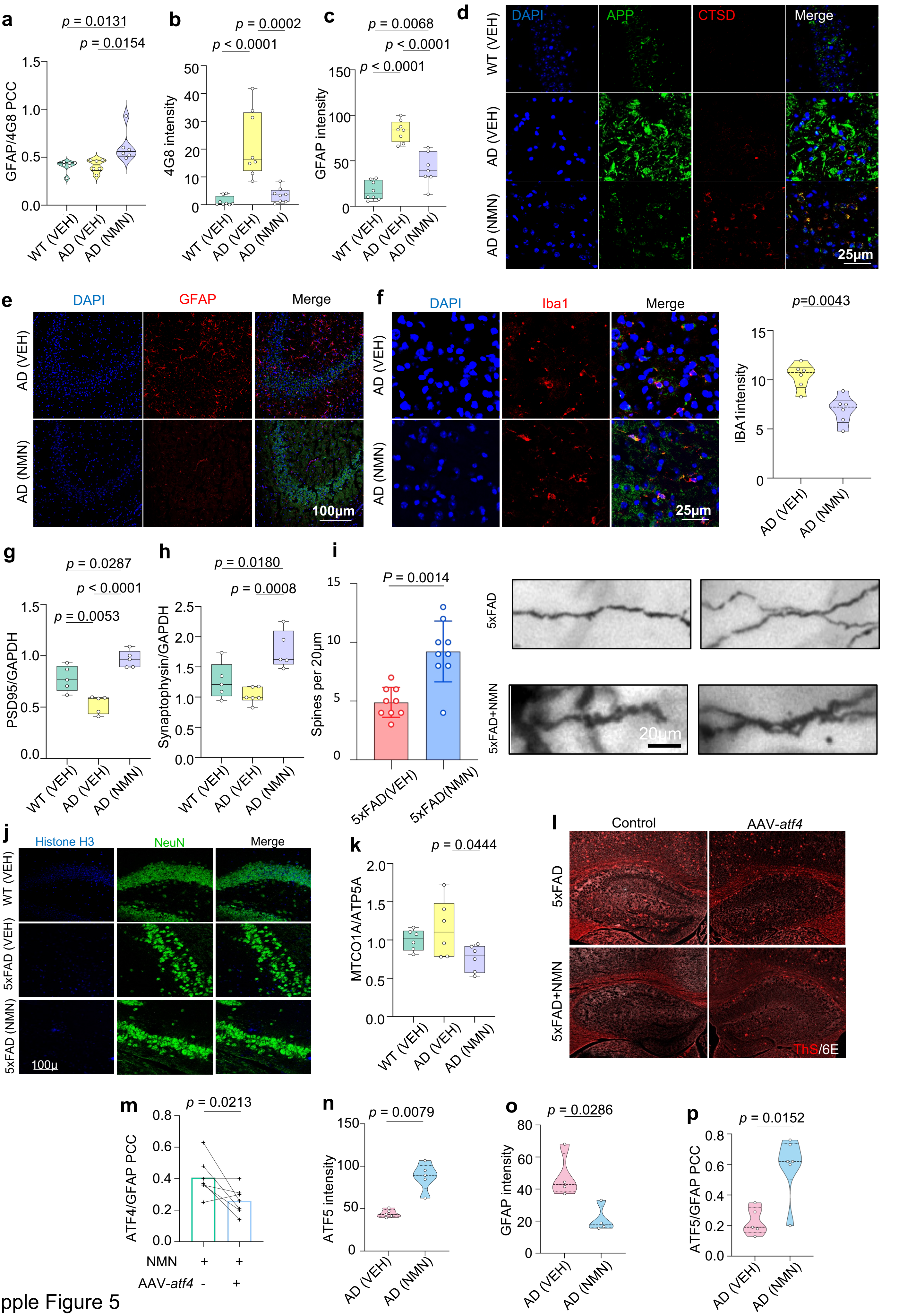

Supple Figure 5

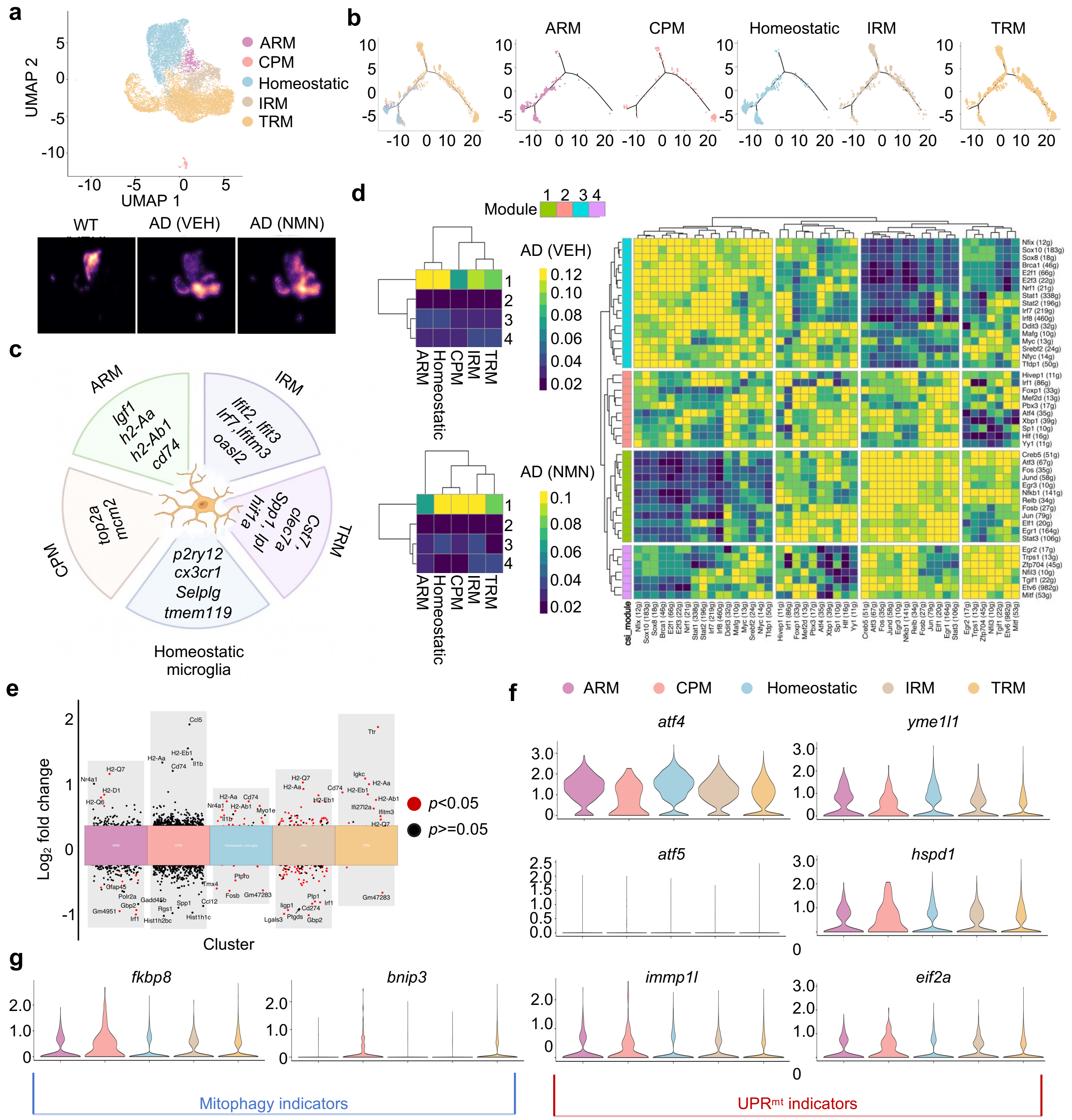

Supple Figure 6

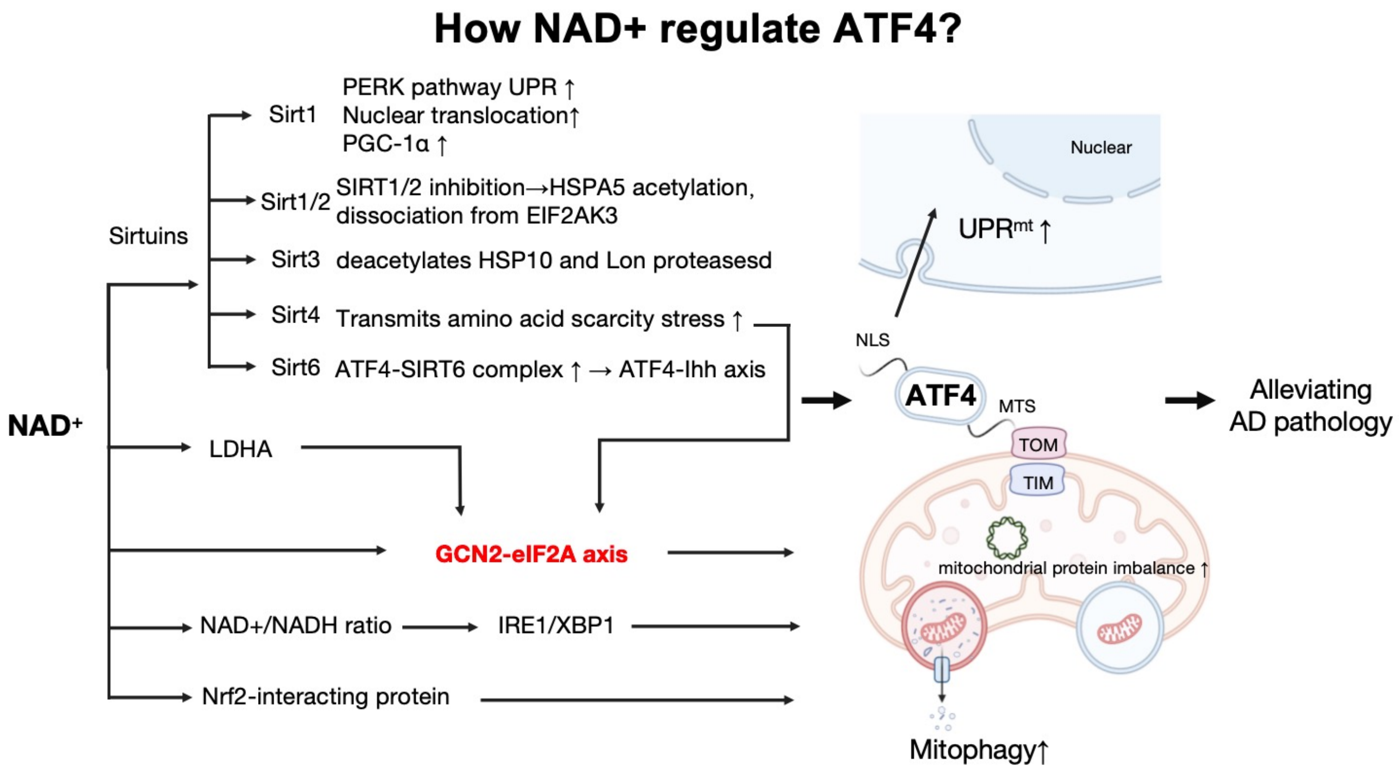

Supple Figure 7

Extended Data Fig. 1 | NMN relieves pathological tau and Aβ deposition in AD cells. a, g, Correlation of disease progression with blood UPR^{mt} and mitophagy biomarker levels in AD patients. **b-e,** Representative images of NMN-supplied N2a APPswe cells stained with anti-β-amyloid$_{1–42}$ and anti-BACE-1 antibody (Scale bar, 25 µm, two-sided unpaired *t*-test). **f,** Transcript levels of the *app* and *bace-1* in both groups. **g,** Representative images indicating the effects of NMN in HEK293 cells Tau P301L. All experiments were performed independently at least twice. Data are shown as mean ± s.e.m. The *p* values are indicated on the graphs. ns, not significant.

Extended Data Fig. 2 | NAD^{+}-UPR^{mt} pathway promotes MSR signature in AD cells. a, d, Representative immunostained images and quantification of ATF5 and CYC in HEK293 cells Tau P301L after being treated with NMN for 24 hours. Scale bar, 10 µm. **b,** Effects of NMN on the expression levels of Hsp70 in NMN-treated N2a APPswe cells after 24h. **c,** Quantification of ATF4 intensity and PCC of ATF4 with DAPI or CYC of N2a APPswe cells after treated with NMN. **e,f,** Representative images and PCC analysis between CHOP and CYC in N2a APPswe cells. **g, i,** The effects of NMN showing increased co-localization between mitophagy (PINK-1) and lysosome (Lamp2) in HEK293 cells Tau P301L. **h,** Graphs show levels of mitophagy-related proteins of NMN-treated N2a APPswe cells in **Fig. 3o**. **j,** Fluorescence intensity and co-localization of autophagy protein LC3 and mitochondrial protein CYC signals which indicate mitophagy events increased in NMN-treated cells. **k,** The mRNA expression measurement of mitophagy genes after supplementing with NMN in N2a APPswe cells (n = 3; two-way ANOVA). Overall fluorescence intensity and PCC differences between groups were assessed by a two-sided unpaired *t*-test. Protein levels were assessed by one-way ANOVA. All experiments were performed independently at least third. Data are shown as mean ± s.e.m. The *p* values are indicated on the graphs. ns, not significant.

Extended Data Fig. 3 | NMN up-regulated anti-oxidation rather tapers apoptosis and **neuroinflammation**. **a-f,** The levels of apoptosis and oxidative stress were detected by Western blotting showing the effects of NMN (n = 3 per group; two-sided one-way ANOVA followed by Tukey's multiple comparisons test). **g-j,** Fluorescence intensity and representative images of Bax and caspase-3 in NMN administration cells (n = 3 per group; two-sided unpaired *t*-test). **k, l,** Representative western blot image of RIPK1, RIPK3, MLKL proteins showing NMN mitigated necroptosis. Data were analyzed by two-sided one-way ANOVA followed by Tukey's multiple comparisons test. *p* values for the associations between mitophagy levels and health outcomes. All experiments were performed independently at least twice. Data are shown as mean ± s.e.m. The *p* values are indicated on the graphs. ns, not significant.

Extended Data Fig. 4 |NAD^{+}-UPR^{mt} signaling ameliorates mitochondrial protein homeostasis through ATF4 *in vitro*. a, Confocal images of supplementation of NMN in Tau P301L cells stained with Mitotracker. Scale bar, 10 µm. **b,** Western blots of mito-nuclear protein imbalance level in N2a APPswe cells. *atf4* knockdown by siRNA in N2a APPswe cell lines was performed by western blotting. **c,** The neuroprotection effect of NMN was dependent on ATF4 and was examined by western blotting and quantified. **d,** Representative images in *atf4 knockdown* N2a APPswe cell lines supplemented with NMN stained with APP antibody. **e,** Knockdown of atf4 aggravated disruption of mito-nuclear protein imbalance and reversed NMN-induced mitophagy. Overall fluorescence intensity and PCC differences between groups were assessed by a two-sided unpaired *t*-test. Protein levels were assessed by one-way ANOVA. All experiments were performed independently at least twice. Data are shown as mean ± s.e.m. The *p* values are indicated on the graphs. ns, not significant.

Extended Data Fig. 5 | NMN attenuates Aβ pathology and neuroinflammation while decreasing synaptic disruption by ATF4-mediated UPR^{mt}. a-c, Quantification of GFAP and 4G8 co-localization and fluorescence intensity of Aβ-positive astrocytes manifesting Aβ plaque were engulfing by astrocytes in NMN- or vehicle-treated 5xFAD mice in Fig 4n. **d**, Immunostaining of APP and lysosome protein CTSD with DAPI in mouse brain sections. **e, f**, Representative immunofluorescence images of GFAP (**e**) and IBA1 (**f**) staining in 6-month-old hippocampi of all genotypes showing the effects of NMN. **g, h**, Quantification of postsynaptic marker PSD-95 and synaptophysin protein level in **5h, i,** Golgi staining and quantification of dendritic spines in the hippocampi of 5xFAD mice. **j**. Representative IF images of all genotypes hippocampal CA1 region showing nuclear histone H3 stained with NeuN. **k**. Quantification of mito-nuclear protein imbalance by MTCO1 and ATP5a ratio in WT, 5xFAD, and 5xFAD (NMN) mice. **l, m**, Representative immunostained images manifesting the effects of NMN in 4G8-positive and ThS-positive amyloid plaques were slashed in the hippocampal and cortex of 6-month AAV atf4 Knowdown 5×FAD mice after a 2-month NMN injection. **n-p**, Semi-quantification of ATF5 and GFAP fluorescence intensity in 5xFAD cultured primary astrocytes after supplementing with NMN for 24h. The colocalization analysis indicates the effects of NMN on enhancing the role of ATF5 in astrocytes. Overall fluorescence intensity and PCC differences between groups were assessed by a two-sided unpaired t-test. Protein levels were assessed by one-way ANOVA. All experiments were performed independently at least twice. Data are shown as mean ± s.e.m. The *p* values are indicated on the graphs. ns, not significant.

Extended Data Fig. 6 | MSR signatures of microglia re-clusters, with two major cell state branches during diversification. a, Re-clustered microglia identifying 5 subclusters (ARM, activated response microglia; CPM, cycling and proliferating microglia; homeostatic microglia; IRM, interferon response microglia; TRM, transiting response microglia). **b,** Cell trajectories diagram of all 5 microglial subcluster cells. We use the Monocle package to implement machine learning to simulate the dynamics of the temporal developmental process of each sub-cluster. Microglia are grouped into three stages (stage 1: ARMs and homeostatic microglia; stage 2: CPMs, IRMs, and TRMs). ARMs and homeostatic microglia were predominantly distributed in the early stage while CPMs, IRMs, and TRMs were in the late stage of the microglia developmental process. **c,** Selected marker genes for re-clustered microglia identification were presented in the graph. **d,** SCENIC analysis shown as a regulons CSI correlation heatmap (right), and heatmap of regulon modules (left) of 5xFAD compared to 5xFAD supplemented with NMN. **e,** Multiple volcano plots demonstrating DEGs in 5 microglia sub-clusters in 5xFAD versus 5xFAD (NMN) samples. **f, g,** Violin plots manifesting the expression of UPR^{mt} marker genes (*atf4, atf5, chop, hspd1, hspd9, eif2a*) and mitophagy marker genes (*binp3l, fkbp8*) in microglia subclusters. All experiments were performed independently at least twice. Data are shown as mean ± s.e.m. The *p* values are indicated on the graphs. ns, not significant.

Extended Data Fig. 7| The potential molecular mechanism on the regulation of ATF4 by NAD$^+$. NAD$^+$ acts through the sirtuins family and most of them are closely related to the GCN2-eIF2α axis. The Sirtuins family can activate the GCN2-eIF2α-ATF4 axis and activate UPR.

Table 1: Basic characteristics of AD patients and healthy controls

Characteristics	HC	AD	p value
	(N = 46)	(N = 43)	
Age (years)	63.804[62.4489,65.1591]	67.302[65.8998,68.7042]	0.0739
Sex:			0.1102
Female	28(60.9%)	33 (76.7%)	
Male	18(39.1%)	10 (23.3%)	
Height (cm)	161.89[160.7022,163.0778]	158.79[157.6236,159.9564]	0.0564
Weight (kg)	63.917[62.5322,65.3018]	53.558[52.1774,54.9386]	p<0.001
BMI (kg/m2)	24.37[23.92978,24.81022]	21.192[20.79493,21.58907]	p<0.001
Education (years)	4.6957[3.94719,5.44421]	4[3.35044,4.64956]	0.7787
Disease History (years)	-	2.9767[2.65494,3.29846]	-
CDR	-	0.9186[0.868877,0.968323]	-
MoCA	-	10.233[9.47063,10.99537]	-
MMSE	24.435[23.84143,25.02857]	15.209[14.1776,16.2404]	p<0.001

Continuous variables were assessed for normality by the Kolmogorov-Smirnov test, P-P plot and Q-Q plot. Data are expressed as median [IQR] (abnormal distribution) or n (%) (categorical variable); P-values obtained from Mann-Whitney U test (abnormal distribution); P-values of categorical variables obtained from chi-squared test. HC: Healthy Control; AD: Alzheimer's disease; BMI: Body Mass Index; CDR: Clinical Dementia Rating; MoCA：Montreal Cognitive Assessment; MMSE: Mini-Mental State Examination.

Table 2: MSR signature biomarkers in AD and HC patients

Blood biomarkers	HC (n = 46)	AD (n = 43)	p value
ATF4(ng/mL)	4.97365[4.97365,5.19015]	4.97365[4.97365,5.19015]	<0.0001
ATF5(ng/mL)	5.66465[5.66465,5.93355]	5.66465[5.66465,5.93355]	<0.0001
CHOP(ng/mL)	9.04544[9.04544,9.49076]	9.04544[9.04544,9.49076]	<0.0001
PINK1(ng/mL)	65.4394[65.4394,68.1046]	87.9187[87.9187,91.9533]	<0.0001
PARKIN(ng/mL)	15.40373[15.40373,16.06627]	20.78281[20.78281,21.76319]	<0.0001

Data are expressed as median [IQR] and *p*-values of were obtained from Mann-Whitney U test.

Table 3: ROC analysis results of MSR signature biomarkers for AD vs HC

Biomarker	AUC	CI	Cut-off value	Sensitivity	Specificity	Accuracy	Youden Index
ATF4	0.956	0.920 - 0.991	6.4998	6.4998	0.7907	0.97826	0.76896
ATF5	0.926	0.875 - 0.976	6.9938	6.9938	0.7907	0.91304	0.70374
CHOP	0.936	0.891 - 0.982	10.401	10.401	1	0.76087	0.76087
PINK1	0.922	0.861 - 0.982	76.823	76.823	0.88372	0.86957	0.75329
Parkin	0.917	0.857 - 0.977	18.582	18.582	0.81395	0.8913	0.70526

AUC: area under roc curve auc.
