# Supplementary material for: NAD+-boosting agent nicotinamide mononucleotide potently improves mitochondria stress response in Alzheimer’s disease via ATF4-dependent mitochondrial UPR
Source: Cell Death Dis. 2024 Oct 11;15(10):744. doi: 10.1038/s41419-024-07062-1 (PMC11470026; doi:10.1038/s41419-024-07062-1)

Western blot full scans

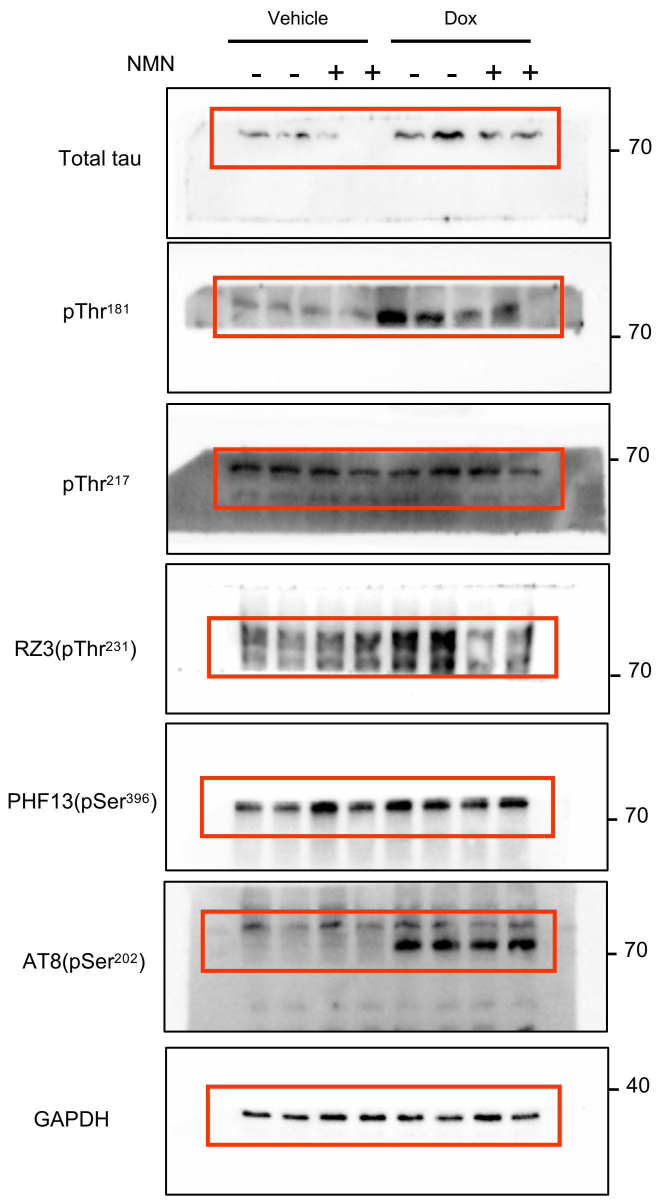

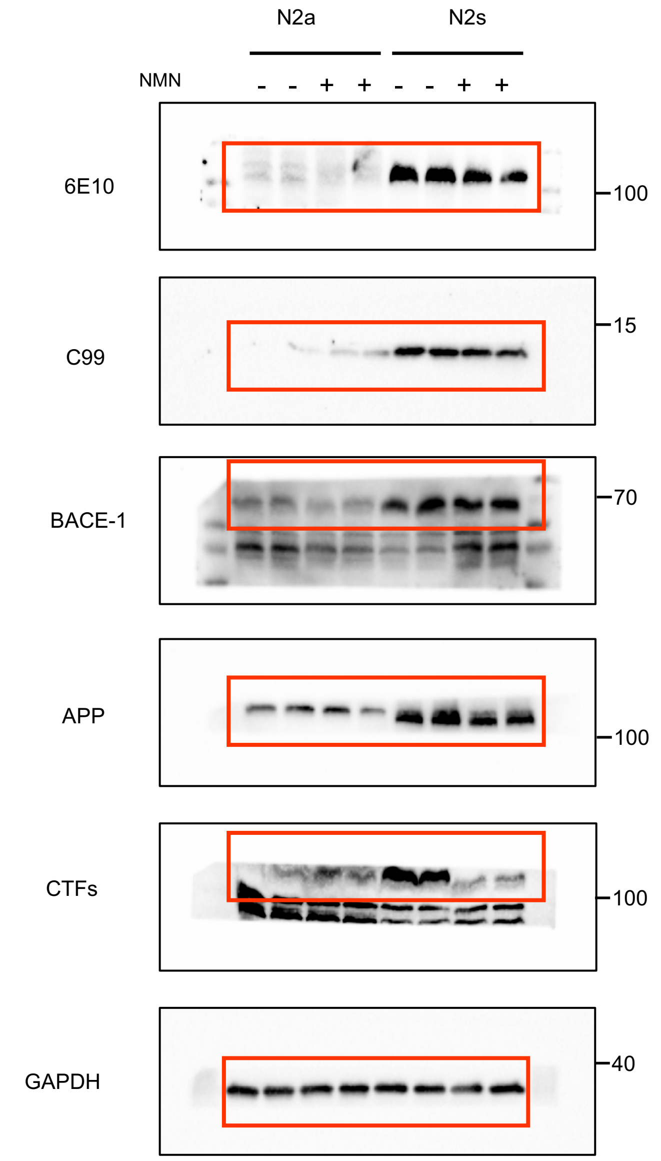

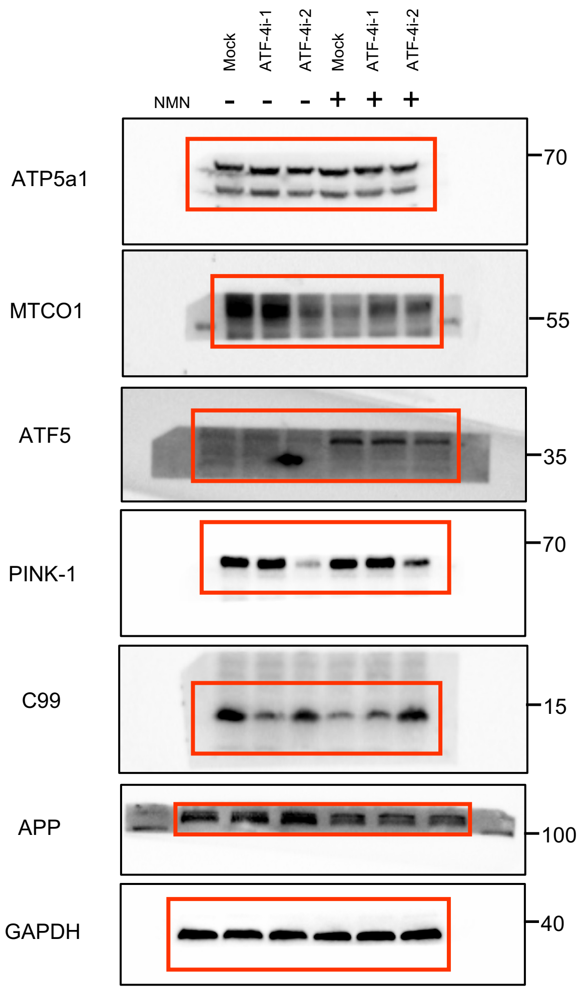

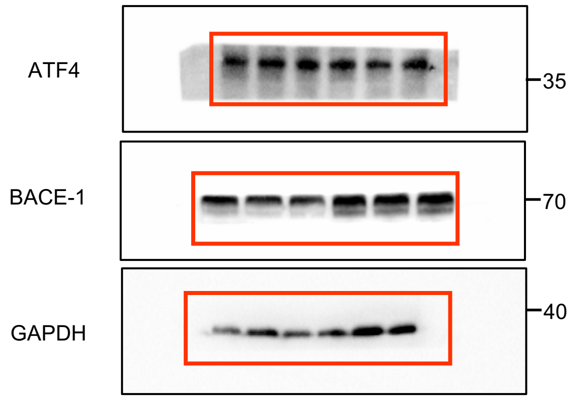

Western blot full scans

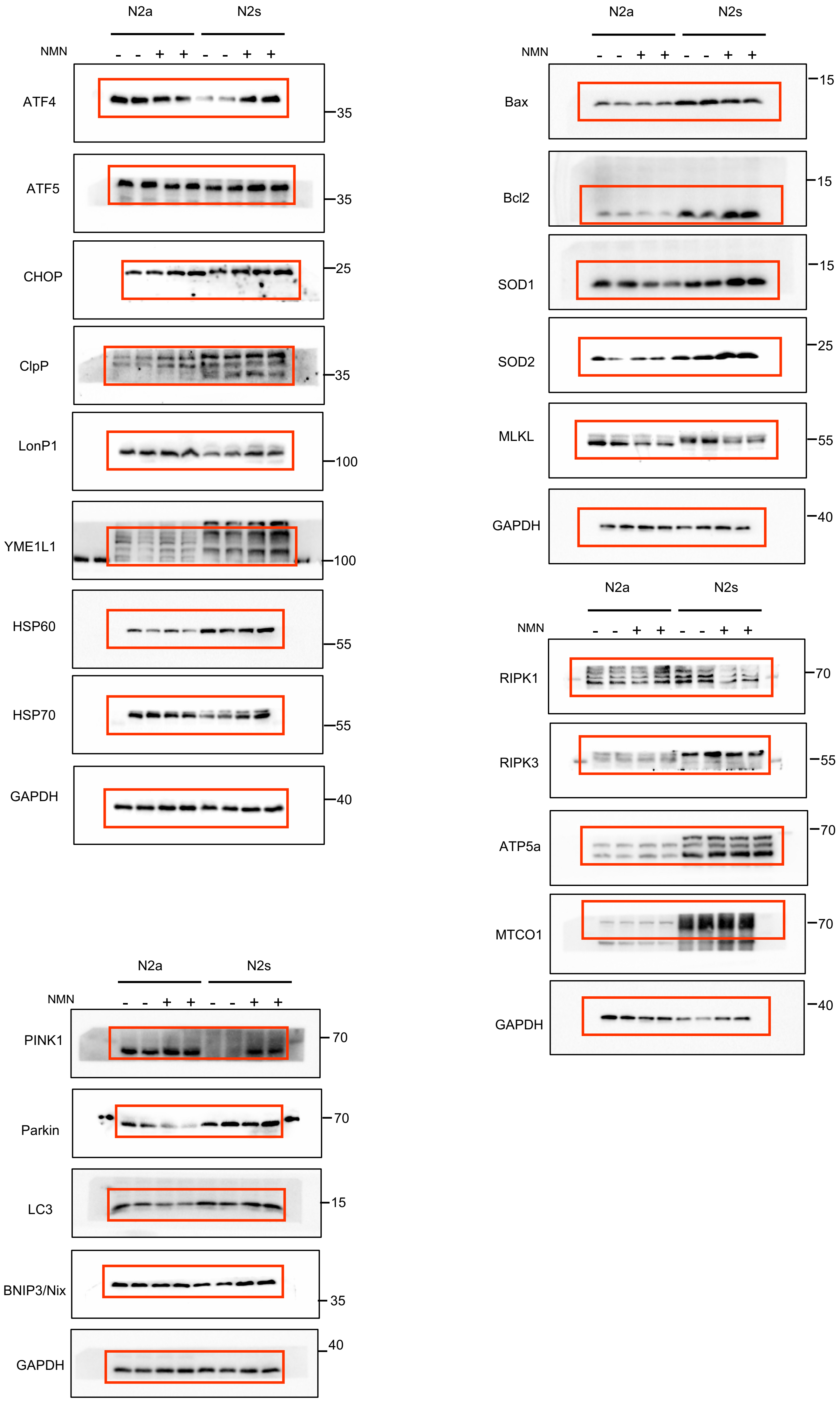

Western blot full scans

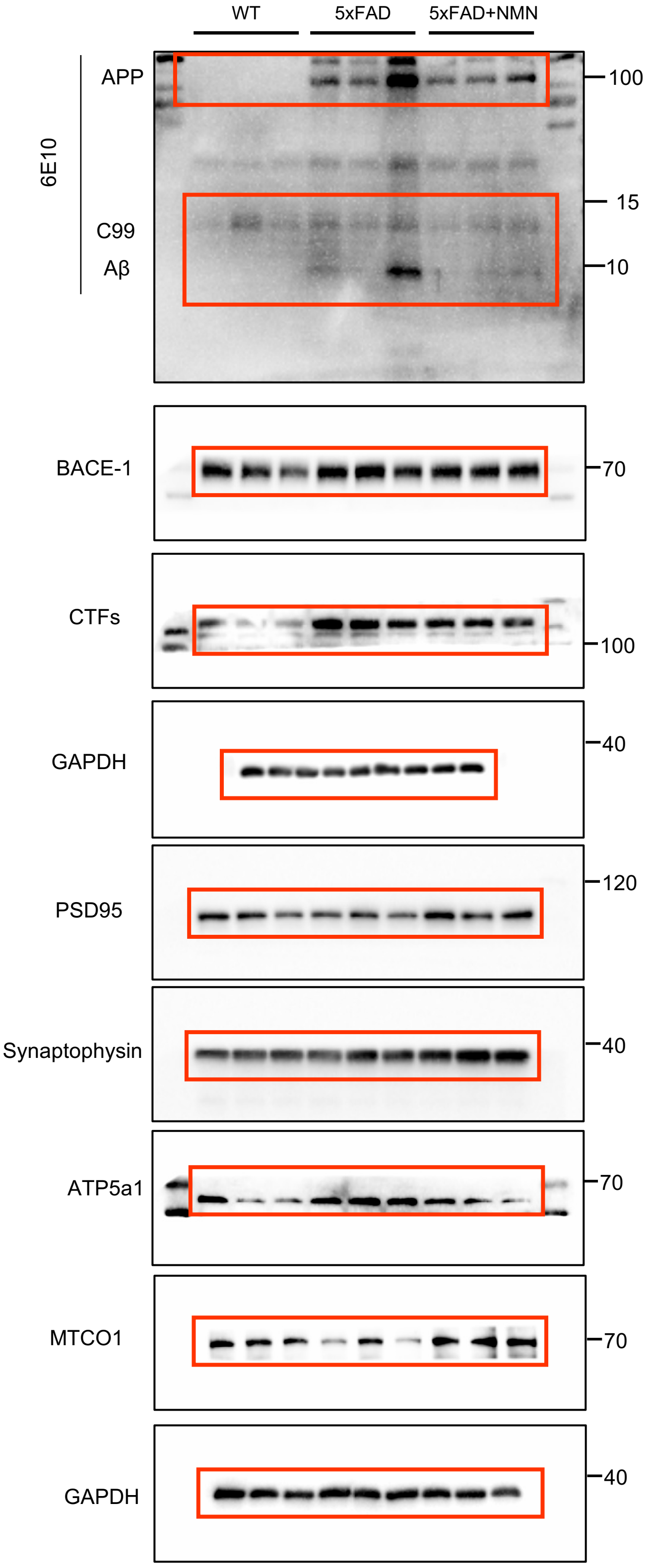

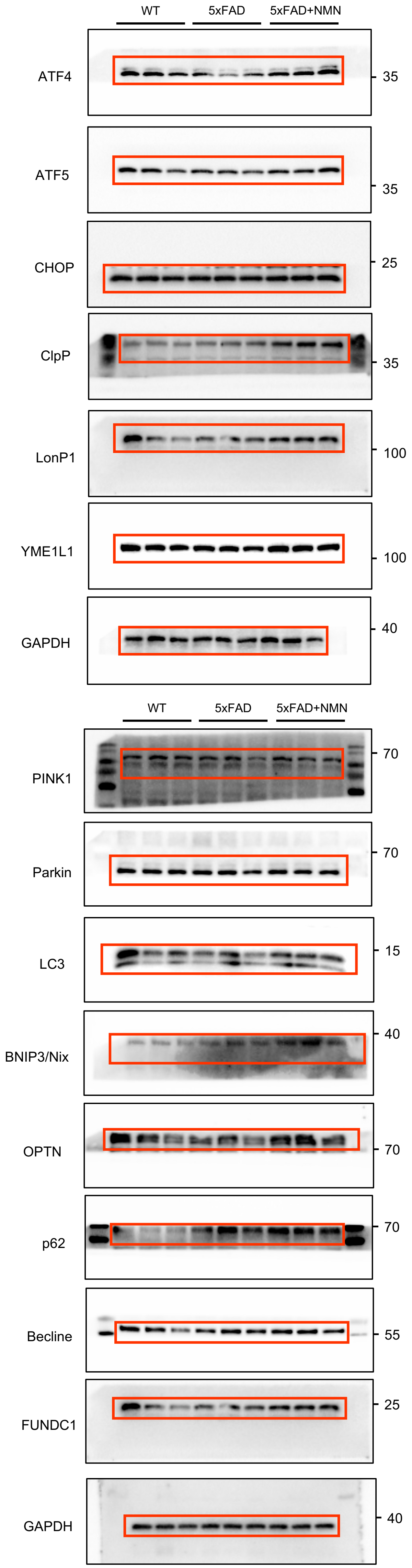

Western blot full scans

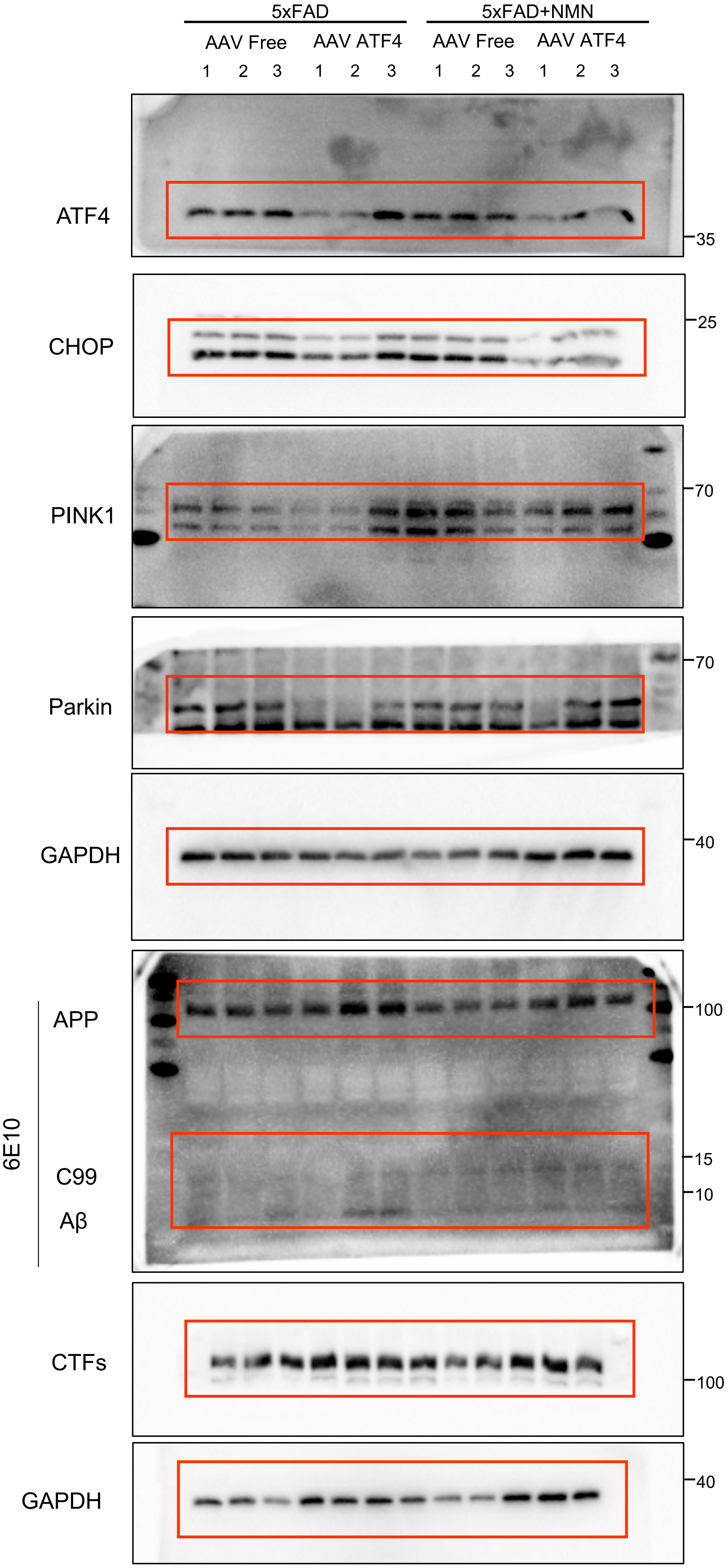

Supplement: Supplementary file 2 — Original Data [file 41419_2024_7062_MOESM2_ESM.pdf]
